# Global burden of pneumoconiosis attributable to occupational particulate matter, gasses, and fumes from 1990~2021 and forecasting the future trends: a population-based study

**DOI:** 10.3389/fpubh.2024.1494942

**Published:** 2025-01-08

**Authors:** Mao Qingsong, Ruijie Xiao, Wenqi Yang, Xinyi Wang, Yu-zhe Kong

**Affiliations:** ^1^Hepatobiliary Pancreatic Surgery, Banan Hospital Affiliated of Chongqing Medical University, Chongqing, China; ^2^Xiangya School of Medicine, Central South University, Changsha, China

**Keywords:** pneumoconiosis, occupational risk, mortality forecasting, epidemiology, disease burden

## Abstract

**Background:**

This study aimed to quantify the global impact of pneumoconiosis resulting from occupational exposure to particulate matter, gasses, and fumes from 1990 to 2021, utilizing data from the Global Burden of Disease Study 2021.

**Method:**

The analysis evaluated the global, regional, and national burden of pneumoconiosis attributable to workplace exposure to particulate matter, gasses, and fumes. It explored variations in disease impact across different demographics, including age and gender, and analyzed the relationship between disease burden and the Socio-Demographic Index (SDI). Furthermore, an ARIMA model was employed to forecast future trends of pneumoconiosis up to 2050.

**Result:**

The year 2021 saw pneumoconiosis from occupational particulate matter, gasses, and fumes account for roughly 4,775 deaths and 117.80 thousand disability-adjusted life years (DALYs). Over the past three decades, there was a notable decline in the disease’s burden. The condition predominantly affected males and those aged above 60. Future projections suggest a decrease in mortality rates in low to middle SDI regions, while high SDI regions may experience an increase in ASMR. Additionally, both ASMR and ASDR are anticipated to rise globally. Nationally, the Czech Republic, France, and the United States are expected to show relatively higher mortality rates in 2030 and 2050. Countries like Kazakhstan, Egypt, Mongolia, and Peru are projected to experience elevated levels of ASMR, DALY rates, and ASDR.

**Conclusion:**

The findings underscore the urgent need for policymakers to create and improve targeted preventive strategies to reduce the incidence of pneumoconiosis among specific populations.

## 1 Introduction

Pneumoconiosis was a chronic occupational disease resulting from prolonged inhalation of solid phase particulate matter, which led to lung dysfunction (1). According to types of occupational dust inhaled, pneumoconiosis was classified into asbestosis, silicosis, coal worker pneumoconiosis (CWP), and other pneumoconiosis (2), especially in developing countries where coal was the main energy source. Millions of workers were regularly exposed to coal dust (including dust from coal mining or pure coal dust) during their working hours, thus having great risk to suffer from pneumoconiosis. Besides, individual behavioral factors such as smoking could be an associated factor due to many cohort studies, indicating that smoking workers had an obviously higher risk than non-smoking workers to develop pneumoconiosis (15). Furthermore, indoor air pollution may contribute to the incidence and exacerbation of hut lung, a domestically acquired pneumoconiosis of mixed etiology (3, 4). Despite decades of prevention efforts, pneumoconiosis remained a global public health problem, constituting a significant portion of occupational diseases (5).

Despite ongoing prevention efforts, coal worker pneumoconiosis (CWP) continues to pose a significant public health challenge globally, representing a substantial fraction of occupational diseases (5). Global Burden of Disease studies indicate a downward trend in pneumoconiosis prevalence since 2015, yet it still affects a significant population, with current figures showing around 527,500 cases and an annual death toll surpassing 21,000 (6–13). Currently, no effective treatment exists for the lung damage caused by pneumoconiosis, with care mostly aimed at symptomatic relief and rehabilitation (1). Understanding and addressing modifiable risk factors is crucial for the global prevention and management of pneumoconiosis.

Risk factors for pneumoconiosis include being male, smoking, older age, and occupational exposure to substances like silica, particulate matter (PM), gasses, fumes, and asbestos (14, 15). Numerous studies confirm the strong correlation between occupational exposures and the development of pneumoconiosis (16, 17). The inhalable fraction of PM can bypass the upper respiratory defenses, damaging lung tissues and cells through mechanisms such as cell apoptosis, oxidative stress, and inflammation, which contribute to the onset of pneumoconiosis (17). Additionally, workers who smoke are at a significantly higher risk of developing pneumoconiosis compared to non-smokers (15). However, a comprehensive evaluation of the impact of occupational particulate matter, gasses, and fumes on pneumoconiosis remains lacking.

Our study analyzed trends in mortality and disability-adjusted life years (DALYs) across different demographics and socio-economic indicators (SDI) from 1990 to 2021, forecasting future trends with the autoregressive integrated moving average (ARIMA) model, drawing on previous research (18, 19). The insights from this study aim to enhance global awareness of occupational hazards like particulate matter, gasses, and fumes, and assist in devising prevention and intervention strategies for coal workers worldwide.

## 2 Method

### 2.1 Study population

Our research utilized data from the 2021 Global Burden of Disease Study, analyzing 369 diseases and injuries and 87 risk factors across 204 countries from 1990 to 2021 (20).

To evaluate the effects of pneumoconiosis, we adopted methodologies from prior studies. Data gathered from various sources such as health surveys and verbal autopsies were refined to increase accuracy. This refined data was analyzed using the Cause of Death Ensemble model (CODEm), which yielded yearly, regional estimates of pneumoconiosis mortality by age and gender. We conducted a comparative risk assessment to identify major risk factors for pneumoconiosis and calculated population attributable fractions (PAF) to assess the impact of occupational hazards on pneumoconiosis rates. These PAFs were crucial for estimating pneumoconiosis-related mortality and disability-adjusted life years (DALYs) across various demographics and periods. DALYs were calculated by summing years lost due to premature death from lower respiratory infections (LRIs) and years lived with disability (YLDs), with adjustments based on disease severity. The Socio-Demographic Index (SDI), formulated from factors such as fertility under age 25 (TFU25), education level for individuals over 15 (EDU15+), and per capita income, classified 204 regions into five SDI categories.

The diagnosis of pneumoconiosis can be found in the https://www.healthdata.org/research-analysis/gbd.

### 2.2 Statistical analysis

**Figure 1:**
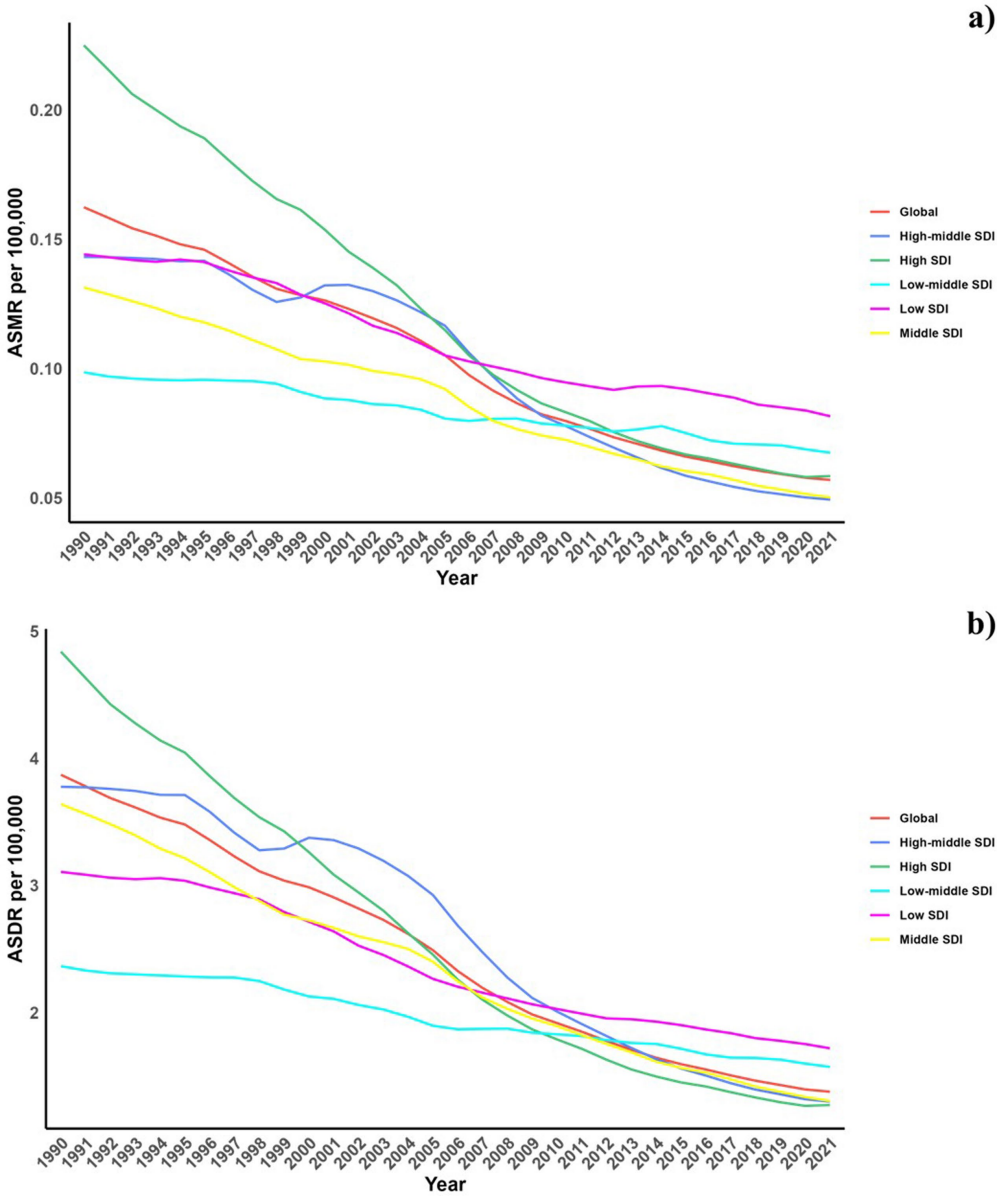
Temporal trends of ASMR and ASDR of Pneumoconiosis attributable to occupational particulate matter, gases, and fumes from1990 to 2021 in different SDI regions.

Age-adjusted rates (AAR) were employed to standardize mortality and DALY statistics across diverse population demographics and age structures. Linear regression was conducted on the natural logs of these measures, modeled as y = *α* + *β*x + *ε*, where x represents the year, and y is the natural log of the rate. The estimated annual percentage change (EAPC) was calculated with the formula 100 * (e^β - 1), including a 95% confidence interval (CI). An increase in AAR was determined if both the EAPC and the lower 95% CI limit were positive, a decrease if both the EAPC and the upper 95% CI limit were negative, and stability if neither condition was met. The relationship between AAR and SDI was examined using Gaussian process regression with Loess smoothing, with the correlation assessed via Spearman rank correlation tests (Figure 1).

Furthermore, an ARIMA (Autoregressive Integrated Moving Average) model was used to study and forecast the impact of low vegetable consumption on pneumoconiosis trends globally, regionally, and nationally from 2020 to 2050. The configuration of the ARIMA model (p, d, q) includes ‘p’ as the number of lag observations in the autoregressive part, ‘q’ as the lag of forecast errors in the moving average component, and ‘d’ as the differencing needed for data stabilization. Model selection was informed by the Akaike Information Criterion (AIC) and the Bayesian Information Criterion (BIC).

All statistical analyses were performed using R. *p* < 0.05 was considered statistically significant (21–25).

## 3 Results

### 3.1 Spatiotemporal patterns of pneumoconiosis attributable to occupational particulate matter, gasses, and fumes

In 2021, exposure to occupational particulate matter, gasses, and fumes resulted in approximately 4,775 deaths and 117.80 thousand disability-adjusted life years (DALYs) due to pneumoconiosis. The age-standardized mortality rate (ASMR) for this condition was 0.0570, with a 95% uncertainty interval (UI) of 0.0487–0.0738, and the age-standardized DALY rate (ASDR) was 1.3771, with a 95% UI of 1.1831–1.7650 per 100,000 people. The data indicate a general decline in the burden of pneumoconiosis from occupational exposures over the last 30 years, as documented in Table 1 and Supplementary Table S1.

**Table 1:** Global and regional deaths and DALYs of pneumoconiosis attributable to occupational particulate matter, gasses, and fumes in 1990 and 2021 in 27 global regions.

Location	Deaths Number in 1990	Deaths Number in 2021	ASMR in 2021	DALY Number in 1990	DALY Number in 2021	ASDR in 2021
Global	6,053 (5,008, 7,371)	4,775 (4,075, 6,210)	0.0570 (0.0738, 0.0487)	156,982 (126,854, 192,041)	117,801 (101,175, 151,164)	1.3771 (1.7650, 1.1831)
Region						
East Asia	2,189 (1,391, 3,096)	1813 (1,306, 3,176)	0.0890 (0.1547, 0.0647)	68,525 (46,056, 94,903)	47,630 (35,670, 79,463)	2.2827 (3.7679, 1.7170)
Southeast Asia	16 (11, 22)	23 (17, 34)	0.0042 (0.0061, 0.0030)	842 (625, 1,093)	1,379 (1,028, 1829)	0.2070 (0.2733, 0.1551)
Oceania	0 (0, 0)	0 (0, 1)	0.0092 (0.0279, 0.0028)	8 (5, 13)	22 (13, 44)	0.3705 (0.7459, 0.2193)
Central Asia	64 (49, 84)	74 (54, 100)	0.0992 (0.1338, 0.0731)	1774 (1,374, 2,278)	2064 (1,577, 2,682)	2.4909 (3.2489, 1.9061)
Central Europe	274 (237, 316)	104 (91, 119)	0.0442 (0.0506, 0.0388)	6,472 (5,622, 7,393)	2,517 (2,222, 2,894)	1.1457 (1.3261, 1.0090)
Eastern Europe	289 (214, 369)	126 (113, 139)	0.0350 (0.0386, 0.0314)	6,689 (4,881, 8,513)	2,942 (2,619, 3,278)	0.8456 (0.9429, 0.7509)
High-income Asia Pacific	367 (316, 428)	524 (433, 629)	0.0962 (0.1169, 0.0786)	9,140 (7,779, 10,587)	9,156 (7,581, 11,073)	1.9653 (2.4030, 1.6371)
Australasia	21 (19, 25)	2 (2, 3)	0.0036 (0.0046, 0.0028)	443 (383, 512)	52 (43, 64)	0.1094 (0.1328, 0.0892)
Western Europe	1,072 (978, 1,163)	240 (211, 259)	0.0204 (0.0219, 0.0181)	21,117 (19,363, 22,887)	3,725 (3,356, 4,024)	0.3642 (0.3956, 0.3294)
Southern Latin America	9 (8, 11)	12 (10, 15)	0.0136 (0.0163, 0.0112)	262 (224, 305)	317 (266, 374)	0.3672 (0.4312, 0.3068)
High-income North America	747 (673, 817)	198 (171, 221)	0.0277 (0.0308, 0.0240)	13,675 (12,513, 14,851)	4,417 (3,867, 5,017)	0.6591 (0.7552, 0.5763)
Caribbean	5 (4, 6)	3 (2, 4)	0.0057 (0.0077, 0.0042)	141 (119, 167)	99 (75, 131)	0.1892 (0.2497, 0.1441)
Andean Latin America	30 (23, 39)	89 (62, 126)	0.1555 (0.2215, 0.1082)	783 (611, 1,014)	1947 (1,392, 2,679)	3.2967 (4.5470, 2.3681)
Central Latin America	65 (61, 70)	104 (90, 119)	0.0438 (0.0499, 0.0378)	1920 (1756, 2,105)	3,269 (2,818, 3,824)	1.3044 (1.5236, 1.1218)
Tropical Latin America	38 (33, 44)	122 (110, 136)	0.0489 (0.0545, 0.0438)	1,326 (1,177, 1,476)	3,296 (2,996, 3,654)	1.2888 (1.4294, 1.1715)
North Africa and Middle East	170 (125, 251)	277 (212, 372)	0.0656 (0.0878, 0.0503)	5,478 (4,264, 7,720)	8,576 (6,657, 11,367)	1.7154 (2.2504, 1.3350)
South Asia	371 (170, 682)	699 (440, 1,041)	0.0545 (0.0818, 0.0342)	10,185 (5,314, 17,397)	17,114 (11,594, 24,771)	1.1755 (1.7208, 0.7875)
Central Sub-Saharan Africa	34 (17, 57)	54 (22, 104)	0.1247 (0.2580, 0.0481)	917 (485, 1,513)	1,446 (624, 2,778)	2.6352 (5.0527, 1.0985)
Eastern Sub-Saharan Africa	96 (48, 169)	109 (61, 172)	0.0772 (0.1208, 0.0434)	2,546 (1,317, 4,475)	2,859 (1,634, 4,624)	1.6511 (2.6216, 0.9455)
Southern Sub-Saharan Africa	36 (27, 51)	38 (29, 62)	0.0755 (0.1226, 0.0578)	948 (731, 1,288)	967 (737, 1,580)	1.6421 (2.6703, 1.2630)
Western Sub-Saharan Africa	159 (83, 262)	164 (105, 281)	0.1042 (0.1770, 0.0673)	3,790 (1952, 6,427)	4,006 (2,589, 6,851)	2.0664 (3.5322, 1.3319)
SDI						
High-middle SDI	1,389 (1,046, 1762)	961 (732, 1,474)	0.0494 (0.0756, 0.0377)	38,507 (29,053, 48,480)	24,695 (19,520, 36,717)	1.2995 (1.9266, 1.0258)
High SDI	2,569 (2,399, 2,725)	1,359 (1,187, 1,564)	0.0585 (0.0678, 0.0510)	54,182 (50,721, 57,584)	26,435 (23,253, 30,413)	1.2723 (1.4780, 1.1155)
Low-middle SDI	530 (349, 806)	871 (656, 1,191)	0.0675 (0.0927, 0.0506)	15,134 (10,302, 21,641)	23,035 (17,795, 30,358)	1.5722 (2.0815, 1.2161)
Low SDI	267 (140, 435)	338 (198, 542)	0.0816 (0.1303, 0.0480)	6,938 (3,725, 11,128)	8,721 (5,307, 13,558)	1.7188 (2.6821, 1.0420)
Middle SDI	1,292 (821, 1887)	1,245 (982, 1964)	0.0502 (0.0790, 0.0398)	42,088 (28,185, 60,441)	34,858 (28,086, 52,370)	1.3049 (1.9534, 1.0524)

Across different Socio-demographic Index (SDI) regions, there has been a reduction in pneumoconiosis burden. Notably, regions with high and high-middle SDI saw a significant drop, whereas the decline was more gradual in low and low-middle SDI areas.

Regionally, the highest number of pneumoconiosis deaths attributable to occupational exposures in 2021 was recorded in East Asia with 1813.2812 (95% UI: 3175.8013–1305.6708) and South Asia with 699.2660 (95% UI: 1041.0488–439.6702), while the lowest were in Australasia at 2.0019 (95% UI: 2.5319–1.5265) and the Caribbean at 3.0230 (95% UI: 4.0778–2.2664). Conversely, Andean Latin America, Central Sub-Saharan Africa, and Western Sub-Saharan Africa experienced the highest burdens in terms of ASMR and ASDR.

**Figure 2:**
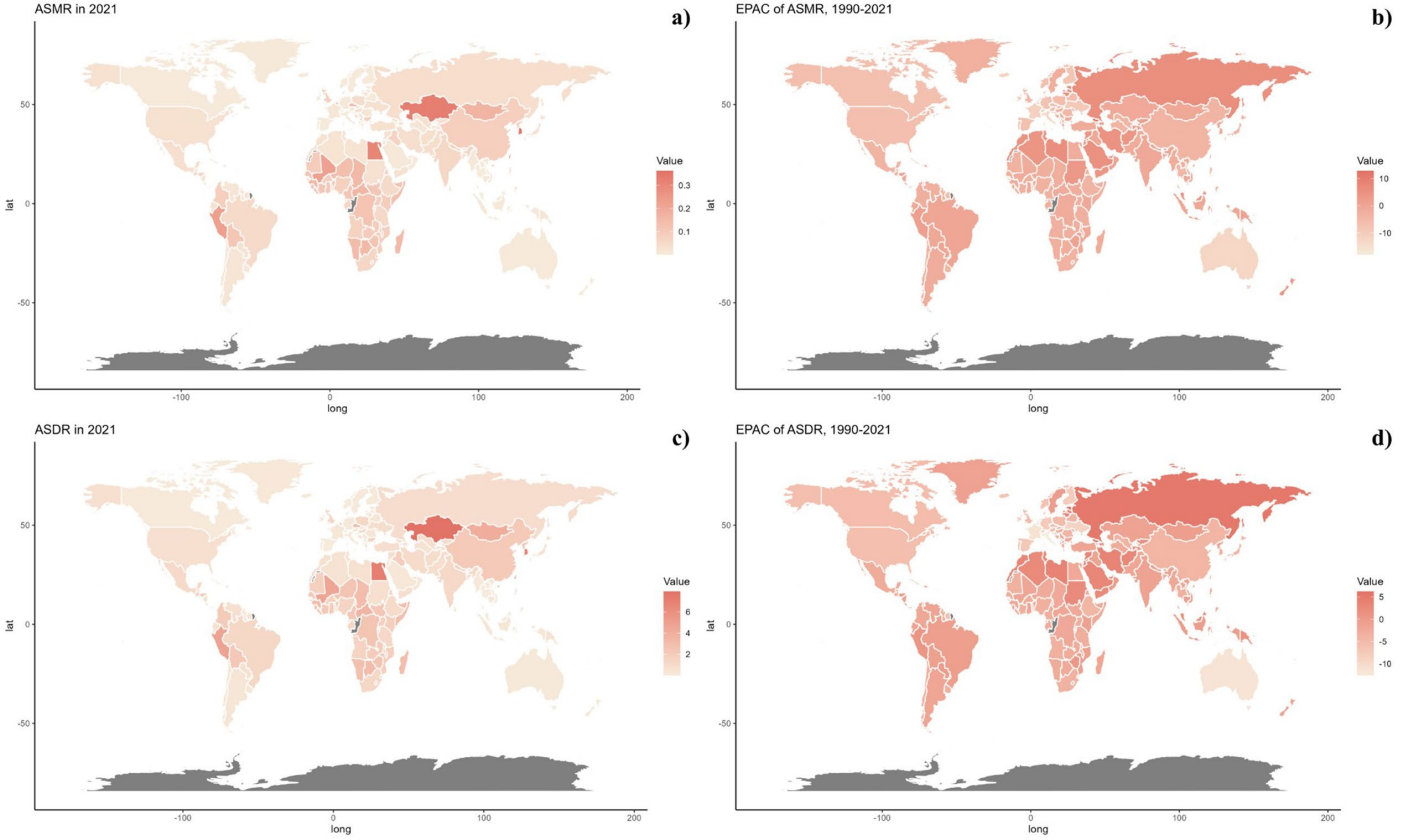
Global distribution of ASMR **(A)** and ASDR **(C)** of pneumoconiosis attributable to occupational particulate matter, gases, and fumes for both sexes in 2021 in 204 countries and territories. EAPC of ASMR **(B)** and ASDR **(D)** of pneumoconiosis attributable to occupational particulate matter, gases, and fumes from 1990 to 2021 in 204 countries and territories.

Nationally, China recorded the highest number of deaths from pneumoconiosis attributable to occupational exposures with 1690.4515 (95% UI: 3044.7846–1186.3571), followed by India with 604.2741 (95% UI: 896.0656–383.4427). Kazakhstan and Egypt reported the highest ASMRs, with Kazakhstan at 0.3288 (95% UI: 0.4798–0.2169) and Egypt at 0.3064 (95% UI: 0.4460–0.2094). Over the past thirty years, nations such as Australia, China, and those in Western Europe and North America have seen substantial reductions in ASMR, while the Russian Federation and Iran have witnessed increases (Table 1; Figure 2; Supplementary Table S1).

### 3.2 Age and gender pattern

**Figure 3:**
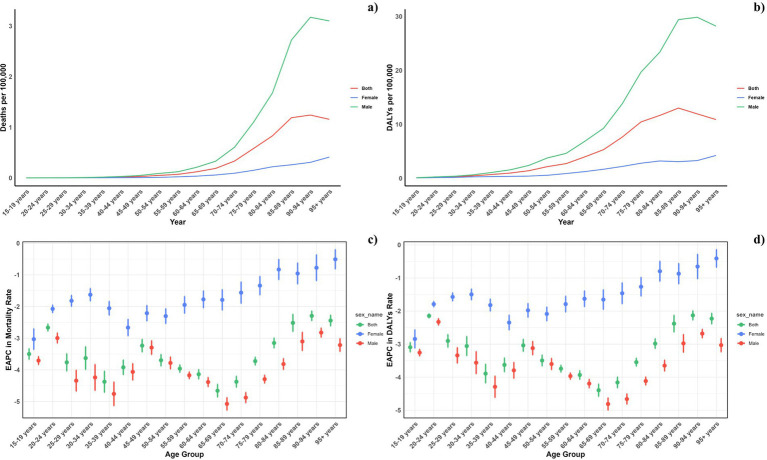
Age-specific rates of global deaths **(A)** and DALYs **(B)** of pneumoconiosis attributable to occupational particulate matter, gases, and fumes, by sex, in 2021 and the corresponding EAPC of global deaths **(C)** and DALYs **(D)** from 1990 to 2021.

**Figure 4:**
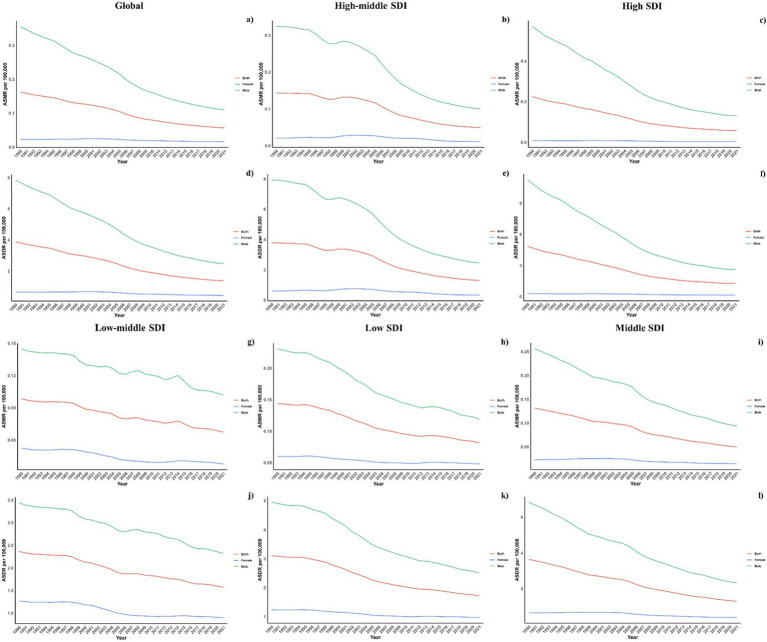
Sex disparity in pneumoconiosis attributable to occupational particulate matter, gases, and fumes across SDI Regions. Note: Global **(a, d)**, high-middle SDI **(b, e)**, high SDI **(c, f)**, low-middle SDI **(g, j)**, low SDI **(h, k)**, middle SDI **(i, l)**; ASMR **(a, b, c, g, h, i)**, ASDR **(d, e, f, j, k, l)**.

Figure 3 illustrated the global mortality and DALY rates for pneumoconiosis across various ages in 2021, as well as trends since 1990. The analysis showed a J-shaped pattern with increasing mortality and DALY rates beginning in the early age groups and escalating sharply from age 60 onwards, reaching peaks in the 90–94 age group for males and 95+ for females. Across all ages, males consistently showed higher mortality and DALY rates compared to females. The estimated annual percentage change (EAPC) varied between −2 and −5, with a steady decrease beginning at age 40–44 for females and 65–69 for males. This gender disparity persisted across all Socio-demographic Index (SDI) regions as illustrated in Figure 4.

### 3.3 Association with the socio-demographic index

**Figure 5:**
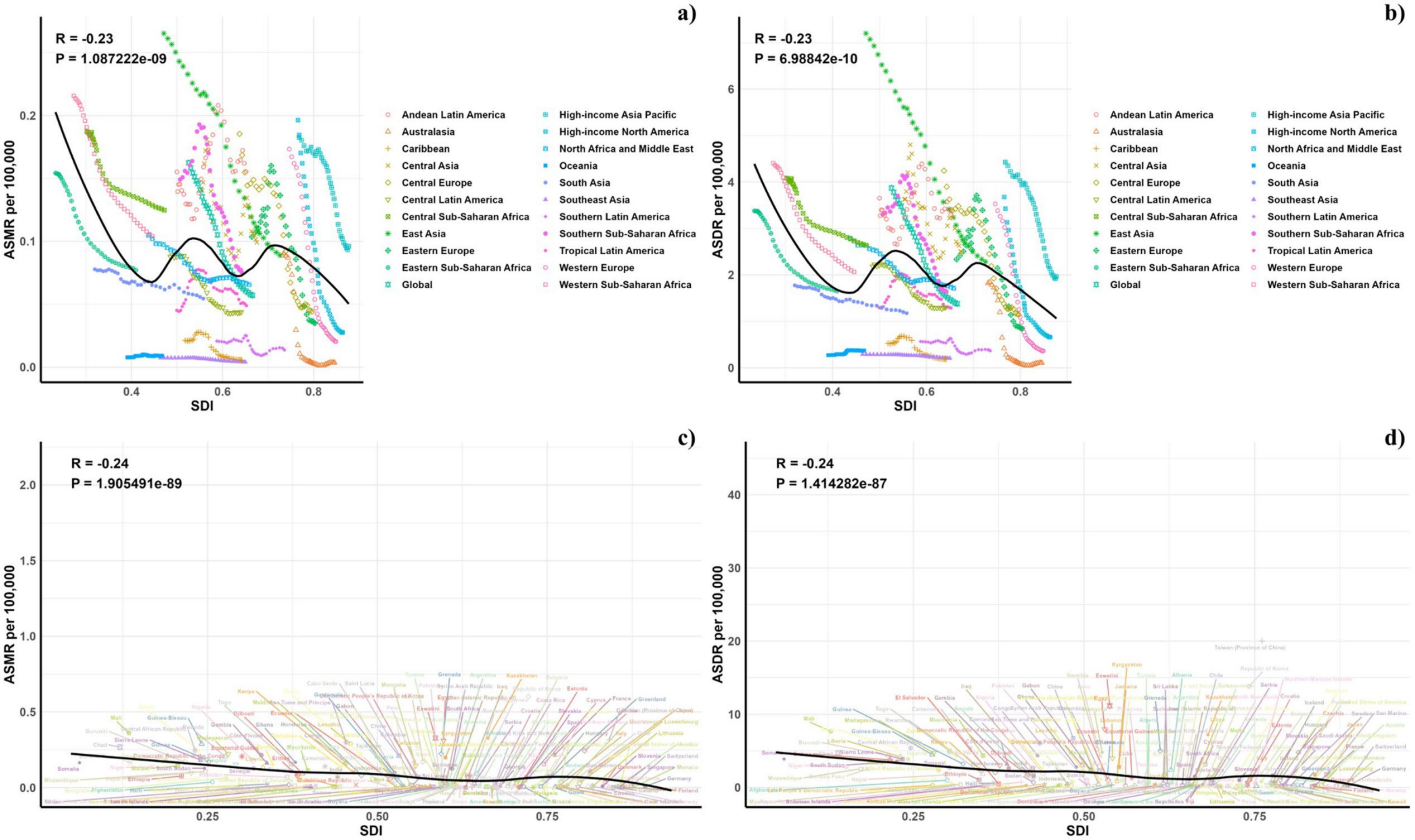
Correlations between ASMR **(A,C)** and ASDR **(B,D)** of pneumoconiosis attributable to occupational particulate matter, gases, and fumes and SDI at the regional level **(A,B)** and the national level **(C,D)**.

Our research evaluated the relationship between the ASDR and ASMR from occupational exposures and SDI values at regional and national levels from 1990 to 2021, as shown in Figure 5. We observed a non-linear relationship between ASDR and SDI (R = −0.23), where ASDR generally declined with increasing SDI but showed fluctuations within middle SDI ranges (0.4 to 0.8). Regions like Western Sub-Saharan Africa, Central Sub-Saharan Africa, Andean Latin America, Central Asia, East Asia, and High-income Asia Pacific reported higher than expected ASDR. Conversely, regions such as Eastern Sub-Saharan Africa, South Asia, Oceania, Tropical Latin America, Southeast Asia, the Caribbean, Southern Latin America, and Australasia had lower than forecasted ASDR. The observed trends in ASMR and ASDR aligned with SDI at regional levels. The 2021 data displayed an inverse relationship between observed and projected ASDR and ASMR at the national level, as depicted in Figure 5.

### 3.4 Forecasts for the mortality, DALYs rate, ASMR, and ASDR of pneumoconiosis attributable to occupational particulate matter, gasses, and fumes in global (2022–2050)

**Figure 6:**
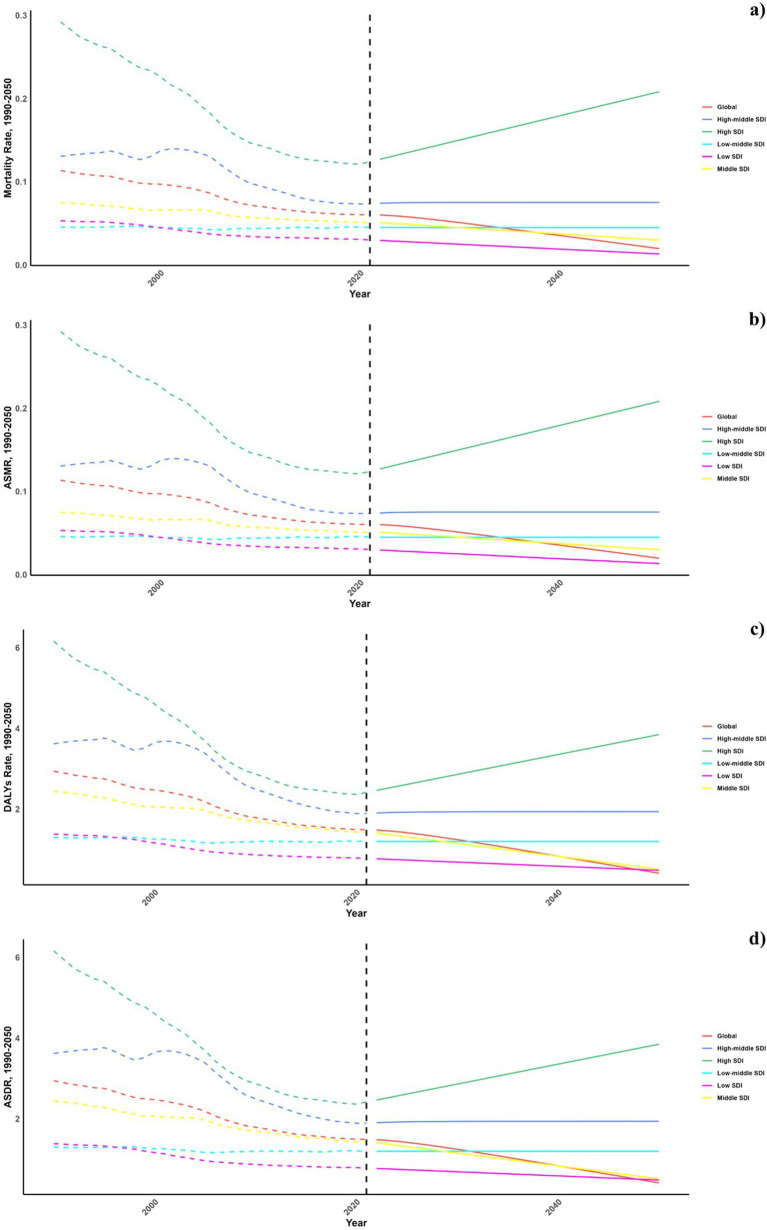
Estimated trends of mortality rate **(A)**, DALYs Rate **(B)**, ASMR **(C)**, and ASDR **(D)**, 1990-2050 at the regional level.

**Figure 7:**
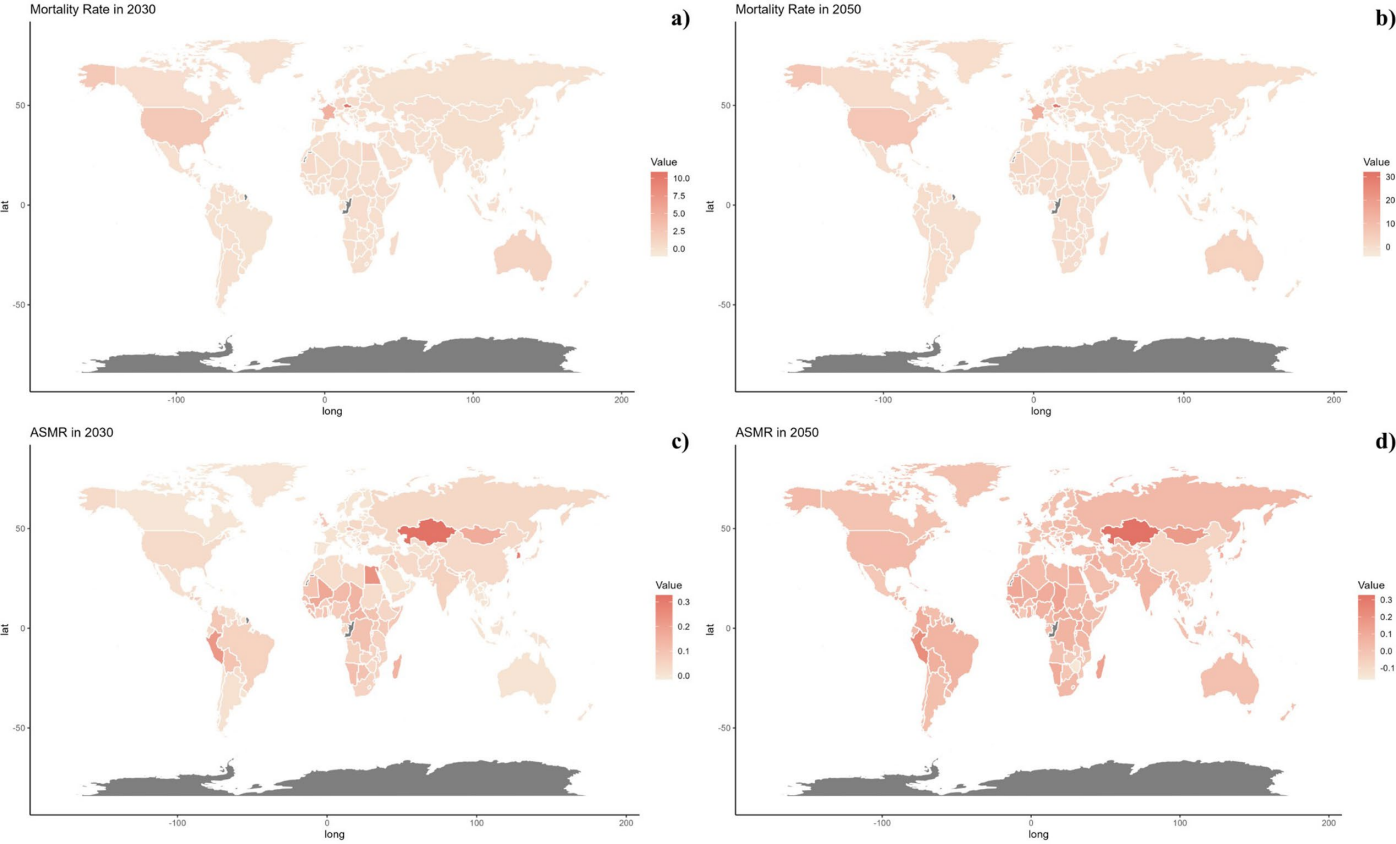
Estimated trends of mortality rate **(A,B)** and ASMR **(C,D)** in 2030 **(A,C)** and 2050 **(B,D)** at the national level.

**Figure 8:**
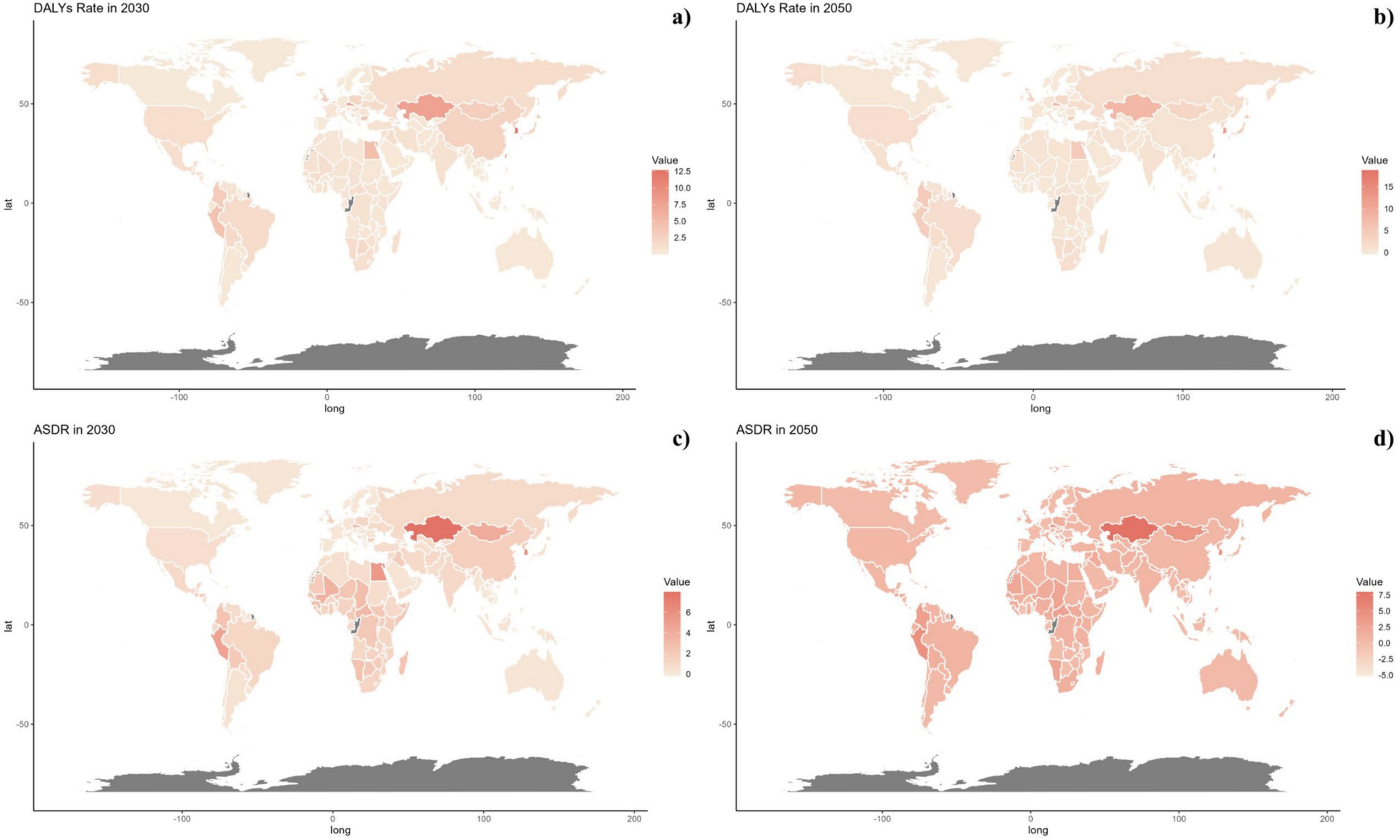
Estimated trends of DALYs Rate **(A,B)** and ASDR **(C,D)** in 2030 **(A,C)** and 2050 **(B,D)** at the national level.

Projections for mortality rates, DALY rates, ASMR, and ASDR related to pneumoconiosis from occupational particulate matter, gasses, and fumes were outlined in Figures 6–8. Forecasts indicate a significant increase in all four indicators in the high SDI regions, whereas a gradual decline is expected in the middle and low SDI regions. The high-middle and low-middle SDI regions are projected to maintain stable rates (Figure 6).

Nationally, projected mortality rates from pneumoconiosis attributable to occupational exposures were notably higher in the Czech Republic, France, and America for both 2030 and 2050. Kazakhstan, Egypt, Mongolia, and Peru are expected to display higher levels of ASMR, DALY rates, and ASDR. It is also significant that ASMR and ASDR are trending upwards globally (Figures 7, 8).

## 4 Discussion

This study was carried out to assess the global burden of pneumoconiosis caused by occupational particulate matter, gasses, and fumes. It comes out that the global mortality rates, DALY rates, ASMR and ASDR of pneumoconiosis showed a declining trend from 1991 to 2021. From a demographic point of view, males and population aged over 60 suffered from pneumoconiosis more seriously. As projections suggested, mortality rates, DALY rates, ASMR, and ASDR would exhibit an increasing trend in the high SDI region while present a decreasing trend in the middle SDI region and low SDI region. In addition, projections also indicate that ASMR and ASDR would be on the rise worldwide. To the best of our knowledge, this study represented the first comprehensive assessment of the impact of pneumoconiosis resulting from occupational particulate matter, gasses, and fumes.

Occupational exposures to asbestos, silica and coal dust (defined as pure coal dust and dust from coal mining) were already recognized as risk factors for pneumoconiosis. As the Global Burden of Disease Study 2016 showed, asbestosis (as a separate outcome from coal workers’ pneumoconiosis and other pneumoconiosis) and silicosis were entirely attributed to occupational exposure to asbestos and silica, respectively, and the burden of coal workers’ pneumoconiosis and other pneumoconiosis was 100% attributable to the risk factors of exposure to occupational particulate matter, gasses, and fumes (2). A cross-sectional study for migrant workers with pneumoconiosis indicated that workers who smoked in the workplace and were exposed to dust were more likely to develop pneumoconiosis (26). Besides, another cohort study subjected on workers exposed to dusts in an iron mine suggested that cumulative exposure dose has an obvious dose-effect relationship with pneumoconiosis incidence (27). Moreover, chronic exposure to high concentrations of fumes during aluminum arc welding could lead to severe pneumoconiosis (28). Besides, benchtop fabrication from quartz conglomerate is also a potentially dangerous occupation. Recent research has highlighted new forms of multiorgan accelerated silicosis could be misdiagnosed as sarcoidosis in workers exposed to quartz conglomerate dust due to atypical clinical presentation (29). Therefore, general practitioners and physicians should have comprehensive understanding of various occupational hazard, especially newly described ones. And accurate occupational history is critical in avoiding misdiagnosis. To sum up, these findings suggested that exposure to particulate matter, gasses, and fumes in the workplace can have significant and lasting effects on lung health, potentially contributing to the onset of pneumoconiosis.

In regard to mechanisms of the pathogenesis of pneumoconiosis, there are already enough studies. Inhaled particle matters were often scavenged by alveolar macrophages, which were activated to produce ROS and RNS, overwhelming the lung’s antioxidant capabilities, resulting in inflammation, which caused disease (30). In response to inhaled PM, airway and alveolar epithelial cells, macrophages produced a variety of cytokines and growth factors, including IL-1β, IL-6, TNF-*α*, macrophage inflammatory proteins (MIP1, MIP2) etc., thus, recruiting other immune cells to the lungs, which produced inflammatory mediators and cell damage (31). Furthermore, some studies have found that accelerated biological aging, as indicated by increased DNA methylation age (DNAmAge) and shortened telomere length (TL), could play a role in patients with lung diseases (32). Additionally, Another research by Plaat et al. implied that occupational exposures might lead to differential methylation of genes involved in regulating gene expression, which could result in other adverse health consequences (32).

In 2017, occupational particulate matter, gasses, and fumes were responsible for 7,000 deaths and 178 thousand DALYs of pneumoconiosis globally, which showed an increase compared to 2007, probably due to population growth in the last decade (33). While age-standardized rates (ASRs) of deaths and DALYs presented a declining trend (33), which might be due to the fact that the prevention policy of pneumoconiosis has been established during these decades. To name a few, in 2016, the Occupational Safety and Health Administration (OSHA) revised standards for occupational exposure to respirable crystalline silica (RCS), limiting permissible exposure to 50 μg/m^3^ and imposing stricter limits on certain industries, which was estimated to reduce health risks significantly. Furthermore, a handful of nations worldwide, namely China, the United States, the United Kingdom, New Zealand, Germany, and Finland, have established robust data collection systems relating to occupational disease, providing more comprehensive data of pneumoconiosis for efficient prevention measures (34). Besides, advancements in dust control technology substantially reduced cumulative dust exposure dose of workers, such as utilizing wet operations to diminish the production and transmission of industrial dust (35), creating a trapezoidal protection area by air curtain to separate the dust-producing surface from the operator (36), etc.

According to the results of our research, ASDR and SDI are negatively correlated roughly. SDI is a composite measure of income per capita, years of education, and total fertility rate. Therefore, this could be due to the fact that areas with low SDI, such as India and Brazil, would likely stem from the poor working conditions, lack of awareness, and underdeveloped healthcare systems prevalent in these regions (37–38), while areas with high SDI possibly had highly developed healthcare systems, high standards of workplace safety procedures, and highly mechanized mining practices that reduced workers’ exposure to particle matters (39). Moreover, we also found that the ASMR and ASDR of pneumoconiosis attributable to occupational particulate matter, gasses, and fumes rose with age in both sexes. This can be attributed to the fact that pneumoconiosis typically had a lengthy latency period, which means that it can take several years or even decades for the disease to develop following exposure to those risk factors (40). The period of exposure to particulate matter, gasses, and fumes that can result in pneumoconiosis may vary based on a range of factors, including the frequency, duration, and intensity of exposure, as well as individual factors such as genetics and overall health. Further research is needed to fully understand the specific mechanisms involved.

In our prediction model, ASMR and ASDR exhibited an increasing trend worldwide, indicating the global burden of pneumoconiosis caused by occupational particulate matter, gasses, and fumes may escalate in the coming decades. However, the International Labor Organization and the World Health Organization were seeking to eradicate this disease by 2030 (41). Therefore, there are still great challenges on the prevention and treatment work of pneumoconiosis. For instance, some countries, like India, had imperfect laws and regulations about the prevention of pneumoconiosis, and other countries, such as China, despite of relevant laws and regulations, there were also problems of lax enforcement of laws and poor supervision (42). Secondly, due to inadequate education and training, some workers lacked basic knowledge of pneumoconiosis and its prevention and control measures (43). Thirdly, many areas lacked regular health checks and early screening measures for pneumoconiosis, and early cases were not detected and treated in a timely manner (43–44). Fourthly, safety measures in the workplace were inadequate. The problem of the shortage of protective equipment and poor ventilation still existed (44–46). As a consequence, policymakers should adopt multiple approaches, including legislation, intensifying supervision, supporting safe work practices, increasing awareness, and offering assistance to affected persons, etc.

Our research confronts several significant constraints. Initially, there is a paucity of primary data from less developed regions, particularly in Sub-Saharan Africa, where evaluations principally rely on mathematical modeling, resulting in extensive variability and uncertainty. Secondly, the statistics regarding pneumoconiosis caused by occupational particulate matter, gasses, and fumes are subject to inconsistencies and biases due to the reliance on self-reports as a data source. Thirdly, detailed quantitative data regarding occupational exposure of workers or occupational categories to particulate matter, gasses, and smoke are lacking.

## 5 Conclusion

This study conducted a detailed analysis of the global impact of pneumoconiosis caused by occupational exposure to particulate matter, gasses, and fumes from 1990 to 2021, showing a notable decline over this period. Men and those over 60 years old bore a higher burden from these exposures. Our forecasts indicate that low to middle SDI regions might experience reductions in mortality rates, whereas high SDI regions could see an uptick in standardized mortality rates. At the country level, projections pointed to particularly high mortality rates in the Czech Republic, France, and the United States for both 2030 and 2050. Additionally, Kazakhstan, Egypt, Mongolia, and Peru are expected to exhibit elevated ASMR, DALY rates, and ASDR.

These findings underscore the critical need for policymakers to craft and enhance specific preventive measures that safeguard and guide workers exposed to hazardous substances for long periods, aiming to reduce the incidence of pneumoconiosis in targeted groups effectively.

## Data Availability

The raw data supporting the conclusions of this article will be made available by the authors, without undue reservation.
