# Supplementary figures and images for: Global burden of pneumoconiosis attributable to occupational particulate matter, gasses, and fumes from 1990~2021 and forecasting the future trends: a population-based study
Source: Front Public Health. 2025 Jan 8;12:1494942. doi: 10.3389/fpubh.2024.1494942 (PMC11751240; doi:10.3389/fpubh.2024.1494942)

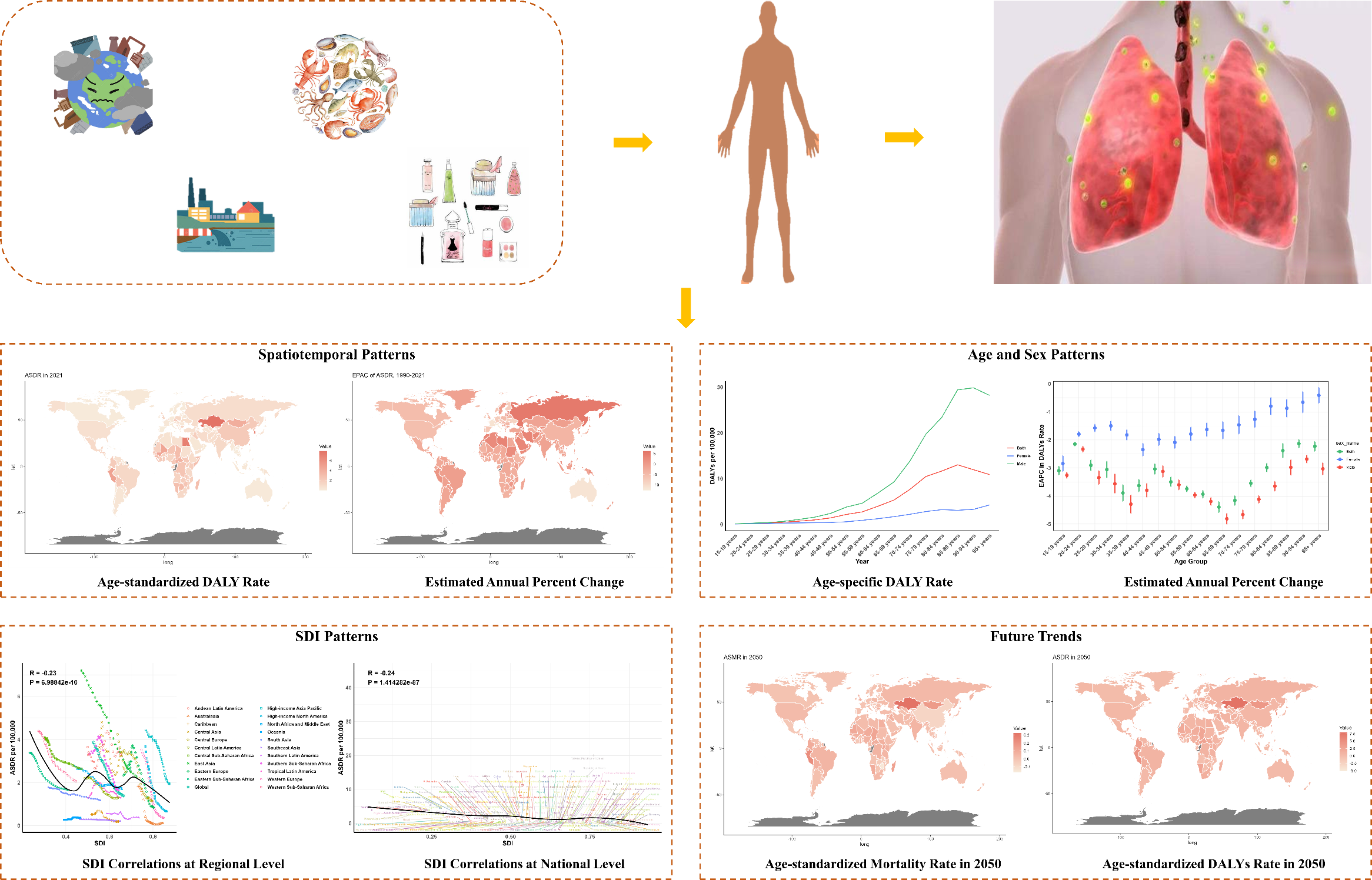

Supplement: Supplementary file 1 [file Data_Sheet_1.docx]
