# Supplementary material for: Global burden of pneumoconiosis attributable to occupational particulate matter, gasses, and fumes from 1990~2021 and forecasting the future trends: a population-based study
Source: Front Public Health. 2025 Jan 8;12:1494942. doi: 10.3389/fpubh.2024.1494942 (PMC11751240; doi:10.3389/fpubh.2024.1494942)
Supplement: Supplementary file 2 [file Table_1.DOCX]

Table S1 Global and regional deaths and DALYs of Pneumoconiosis Attributable to Occupational Particulate Matter, Gases, and Fumes in 1990 and 2021 in 204 nations

| Location | Deaths Number in 1990 | Deaths Number in 2021 | ASMR in 2021 | DALY Number in 1990 | DALY Number in 2021 | ASDR in 2021 |
| --- | --- | --- | --- | --- | --- | --- |
| China | 2117.4603 (3025.0438, 1321.3445) | 1690.4515 (3044.7846, 1186.3571) | 0.0859 (0.1540, 0.0606) | 66301.6091 (92592.2627, 43954.9445) | 44532.4312 (76632.2524, 32531.2573) | 2.2099 (3.7643, 1.6227) |
| Democratic People's Republic of Korea | 14.4232 (23.2519, 5.6146) | 21.4717 (43.5588, 7.1293) | 0.0651 (0.1322, 0.0220) | 551.7645 (844.8274, 265.9610) | 760.0203 (1488.8080, 333.2373) | 2.2713 (4.4112, 1.0021) |
| Taiwan (Province of China) | 56.7889 (65.0557, 48.2413) | 101.3580 (118.9946, 85.0406) | 0.2404 (0.2814, 0.2017) | 1671.9510 (1894.1229, 1438.4761) | 2337.5479 (2677.3088, 1998.5840) | 5.7763 (6.5957, 4.9734) |
| Cambodia | 0.2074 (0.3318, 0.1244) | 0.4953 (0.8628, 0.2724) | 0.0056 (0.0100, 0.0032) | 11.2945 (15.5375, 7.8237) | 23.3722 (33.1629, 16.2084) | 0.1875 (0.2679, 0.1297) |
| Indonesia | 4.5943 (6.5984, 3.0155) | 8.4633 (13.7746, 5.3582) | 0.0046 (0.0077, 0.0030) | 437.0709 (606.3622, 306.7867) | 788.9247 (1080.3020, 572.4496) | 0.3114 (0.4220, 0.2255) |
| Lao People's Democratic Republic | 0.1456 (0.2423, 0.0773) | 0.1935 (0.3542, 0.1021) | 0.0052 (0.0102, 0.0027) | 6.6436 (9.4593, 4.3205) | 9.4331 (13.7288, 6.2554) | 0.1878 (0.2754, 0.1242) |
| Malaysia | 0.0626 (0.0997, 0.0374) | 0.4070 (0.5768, 0.2776) | 0.0017 (0.0024, 0.0011) | 10.6986 (15.5155, 7.1110) | 38.9622 (54.0542, 26.5848) | 0.1330 (0.1830, 0.0925) |
| Maldives | 0.1496 (0.2079, 0.0903) | 0.1938 (0.2810, 0.1343) | 0.0644 (0.0924, 0.0445) | 4.9279 (6.9357, 3.0416) | 5.6065 (8.0258, 3.9767) | 1.4612 (2.0792, 1.0398) |
| Myanmar | 2.4896 (4.2467, 1.3427) | 3.3074 (5.5898, 1.8387) | 0.0085 (0.0150, 0.0047) | 96.3090 (142.5862, 62.1488) | 112.7487 (164.2042, 76.0157) | 0.2396 (0.3549, 0.1618) |
| Philippines | 3.1876 (3.9229, 2.6306) | 3.2311 (4.3178, 2.5556) | 0.0049 (0.0065, 0.0039) | 86.1197 (106.2592, 70.9621) | 112.8225 (143.2226, 91.0335) | 0.1372 (0.1732, 0.1108) |
| Sri Lanka | 1.5545 (2.2608, 1.0892) | 1.7882 (3.1361, 0.9974) | 0.0076 (0.0136, 0.0043) | 51.3787 (69.3476, 37.6711) | 62.9195 (90.9558, 43.1403) | 0.2448 (0.3496, 0.1695) |
| Thailand | 1.1436 (1.8453, 0.5753) | 1.1440 (1.8889, 0.5996) | 0.0011 (0.0018, 0.0006) | 35.3779 (54.1748, 21.3180) | 38.4250 (56.1740, 26.3108) | 0.0387 (0.0570, 0.0267) |
| Timor-Leste | 0.0142 (0.0236, 0.0073) | 0.0389 (0.0687, 0.0204) | 0.0055 (0.0098, 0.0029) | 0.8076 (1.1702, 0.5165) | 1.7771 (2.5883, 1.1765) | 0.1975 (0.2829, 0.1327) |
| Viet Nam | 2.0103 (3.6755, 0.9987) | 3.3708 (5.9409, 1.7891) | 0.0042 (0.0074, 0.0023) | 98.6876 (140.9269, 68.7565) | 179.1172 (251.0912, 124.9909) | 0.1854 (0.2577, 0.1296) |
| Fiji | 0.0016 (0.0041, 0.0005) | 0.0023 (0.0057, 0.0007) | 0.0007 (0.0018, 0.0002) | 0.5959 (0.8976, 0.3840) | 1.0165 (1.5016, 0.6462) | 0.1412 (0.2049, 0.0912) |
| Kiribati | 0.0015 (0.0042, 0.0005) | 0.0029 (0.0081, 0.0009) | 0.0091 (0.0284, 0.0023) | 0.0949 (0.1545, 0.0592) | 0.2191 (0.3566, 0.1358) | 0.4363 (0.7228, 0.2604) |
| Marshall Islands | 0.0006 (0.0012, 0.0003) | 0.0010 (0.0021, 0.0004) | 0.0059 (0.0132, 0.0025) | 0.0396 (0.0567, 0.0270) | 0.0727 (0.1076, 0.0479) | 0.2923 (0.4239, 0.1934) |
| Micronesia (Federated States of) | 0.0024 (0.0054, 0.0010) | 0.0028 (0.0066, 0.0011) | 0.0077 (0.0196, 0.0028) | 0.1246 (0.1896, 0.0805) | 0.1601 (0.2378, 0.1071) | 0.2905 (0.4606, 0.1887) |
| Papua New Guinea | 0.1028 (0.2991, 0.0327) | 0.3607 (1.2901, 0.0897) | 0.0131 (0.0407, 0.0034) | 5.4264 (10.1574, 3.2117) | 17.2312 (37.4303, 9.7593) | 0.4606 (0.9948, 0.2518) |
| Samoa | 0.0027 (0.0070, 0.0009) | 0.0048 (0.0123, 0.0017) | 0.0049 (0.0133, 0.0017) | 0.1874 (0.2832, 0.1185) | 0.3422 (0.5041, 0.2236) | 0.2673 (0.4040, 0.1748) |
| Solomon Islands | 0.0031 (0.0062, 0.0014) | 0.0101 (0.0300, 0.0036) | 0.0055 (0.0152, 0.0018) | 0.2999 (0.4321, 0.2022) | 0.8200 (1.3424, 0.5263) | 0.2944 (0.4741, 0.1853) |
| Tonga | 0.0015 (0.0036, 0.0007) | 0.0029 (0.0069, 0.0013) | 0.0042 (0.0105, 0.0018) | 0.1070 (0.1530, 0.0703) | 0.1876 (0.2792, 0.1307) | 0.2423 (0.3609, 0.1672) |
| Vanuatu | 0.0007 (0.0020, 0.0002) | 0.0018 (0.0068, 0.0004) | 0.0020 (0.0073, 0.0004) | 0.1295 (0.1898, 0.0832) | 0.3593 (0.5265, 0.2301) | 0.2478 (0.3666, 0.1525) |
| Armenia | 0.8533 (1.1949, 0.5695) | 0.7720 (0.9846, 0.5986) | 0.0180 (0.0230, 0.0141) | 29.5351 (38.8346, 21.5467) | 25.4379 (31.4959, 20.7231) | 0.6118 (0.7596, 0.4947) |
| Azerbaijan | 0.8132 (1.6021, 0.3746) | 2.1279 (4.1422, 1.0709) | 0.0250 (0.0473, 0.0136) | 30.3028 (48.4819, 18.3983) | 73.2037 (132.6552, 42.0421) | 0.7240 (1.2597, 0.4276) |
| Georgia | 0.3000 (0.3833, 0.2344) | 2.5013 (3.2196, 1.8946) | 0.0410 (0.0535, 0.0307) | 23.0690 (31.1283, 16.7282) | 69.3307 (88.8708, 53.6112) | 1.2012 (1.5389, 0.9222) |
| Kazakhstan | 48.9943 (68.2382, 35.1361) | 55.0694 (80.5033, 36.4076) | 0.3288 (0.4798, 0.2169) | 1320.7555 (1802.6640, 967.7355) | 1457.2117 (2092.5303, 994.6462) | 7.9347 (11.3854, 5.4040) |
| Kyrgyzstan | 0.6910 (1.3243, 0.3940) | 1.4414 (2.4713, 0.9302) | 0.0331 (0.0602, 0.0207) | 24.1294 (37.3251, 15.9659) | 48.3498 (74.9134, 34.5347) | 0.9531 (1.5148, 0.6804) |
| Mongolia | 1.8088 (3.0550, 0.9502) | 3.4441 (5.2023, 2.1917) | 0.1672 (0.2584, 0.1042) | 51.1832 (80.1178, 30.1736) | 102.7962 (146.8874, 69.3654) | 4.0870 (5.9208, 2.7170) |
| Tajikistan | 0.2969 (0.5959, 0.1361) | 0.8519 (1.7710, 0.3709) | 0.0210 (0.0412, 0.0092) | 13.0810 (19.7212, 8.4825) | 33.1188 (54.0668, 19.7191) | 0.5957 (0.9886, 0.3579) |
| Turkmenistan | 0.6947 (1.0670, 0.4203) | 0.5252 (0.8790, 0.3117) | 0.0138 (0.0230, 0.0083) | 22.7834 (32.7782, 15.4405) | 23.9775 (35.6928, 16.5644) | 0.5542 (0.8158, 0.3821) |
| Uzbekistan | 9.5798 (13.8292, 7.0659) | 7.1318 (9.3166, 5.3003) | 0.0325 (0.0426, 0.0238) | 259.2446 (355.0644, 199.1880) | 230.3179 (287.5843, 179.7079) | 0.8839 (1.1084, 0.6860) |
| Albania | 2.9084 (5.4312, 1.3323) | 3.7877 (7.6952, 1.6355) | 0.0878 (0.1769, 0.0384) | 68.5655 (119.5167, 36.2985) | 79.6288 (147.8456, 40.2043) | 1.8143 (3.3432, 0.9276) |
| Bosnia and Herzegovina | 2.6378 (4.9100, 1.3217) | 2.6273 (4.6103, 1.4193) | 0.0406 (0.0708, 0.0222) | 69.0994 (117.0624, 41.0860) | 63.1568 (98.2491, 40.4163) | 1.0205 (1.5817, 0.6594) |
| Bulgaria | 9.7445 (12.2404, 7.8587) | 11.1821 (15.4913, 7.9413) | 0.0762 (0.1042, 0.0548) | 239.3302 (298.0068, 196.2062) | 235.4386 (311.4262, 174.6394) | 1.6816 (2.2059, 1.2660) |
| Croatia | 1.3177 (1.6881, 1.0165) | 0.9769 (1.4336, 0.6675) | 0.0098 (0.0143, 0.0068) | 32.0631 (39.0714, 25.9078) | 24.7403 (32.1091, 18.9717) | 0.2830 (0.3643, 0.2168) |
| Czechia | 141.2565 (178.5844, 110.9559) | 33.3083 (42.7561, 24.9609) | 0.1444 (0.1851, 0.1079) | 3165.6094 (3978.2245, 2475.3523) | 716.7047 (912.4576, 548.3316) | 3.3256 (4.2214, 2.5370) |
| Hungary | 17.2420 (24.8759, 11.8931) | 7.8332 (10.2731, 5.9549) | 0.0376 (0.0495, 0.0285) | 414.9061 (588.3220, 297.2096) | 189.5240 (238.1101, 149.2566) | 0.9900 (1.2519, 0.7760) |
| North Macedonia | 0.1166 (0.2837, 0.0593) | 0.2324 (0.4558, 0.1009) | 0.0090 (0.0167, 0.0040) | 3.1035 (6.5446, 1.8296) | 6.1565 (10.6587, 3.4738) | 0.2083 (0.3448, 0.1198) |
| Montenegro | 0.1079 (0.2154, 0.0459) | 0.1280 (0.2821, 0.0517) | 0.0146 (0.0320, 0.0059) | 2.7316 (5.1045, 1.4125) | 2.9437 (5.5241, 1.5458) | 0.3172 (0.5868, 0.1725) |
| Poland | 75.1141 (83.7351, 67.6124) | 31.2705 (36.8963, 26.4776) | 0.0418 (0.0494, 0.0354) | 1918.5382 (2142.1358, 1729.7635) | 902.0976 (1057.3276, 760.4840) | 1.2935 (1.5181, 1.0868) |
| Romania | 11.4192 (16.5244, 7.6525) | 5.8856 (8.2452, 4.1296) | 0.0145 (0.0201, 0.0103) | 254.0070 (353.6755, 182.3732) | 123.7242 (163.5553, 92.3565) | 0.3344 (0.4341, 0.2482) |
| Serbia | 2.4745 (3.8205, 1.6021) | 2.1649 (3.2994, 1.3624) | 0.0124 (0.0188, 0.0080) | 61.6255 (93.4866, 43.3406) | 53.8197 (75.1643, 38.4137) | 0.3297 (0.4542, 0.2377) |
| Slovakia | 4.3092 (8.0601, 2.7939) | 2.6759 (4.1173, 1.5629) | 0.0275 (0.0422, 0.0163) | 114.2147 (197.1794, 77.6351) | 72.6645 (103.2487, 50.7910) | 0.7852 (1.1051, 0.5554) |
| Slovenia | 1.0918 (1.3923, 0.8523) | 0.4433 (0.5738, 0.3410) | 0.0086 (0.0110, 0.0066) | 24.9471 (31.0914, 19.5300) | 9.5787 (11.9749, 7.5996) | 0.2101 (0.2640, 0.1670) |
| Belarus | 4.1089 (5.6553, 2.6408) | 1.0834 (1.4768, 0.7578) | 0.0067 (0.0089, 0.0047) | 122.8762 (162.6776, 90.4028) | 54.9061 (76.0645, 41.3144) | 0.3541 (0.4864, 0.2647) |
| Estonia | 0.0202 (0.0267, 0.0148) | 0.2353 (0.3014, 0.1771) | 0.0075 (0.0097, 0.0056) | 0.8749 (1.1599, 0.6337) | 7.0171 (8.9698, 5.4072) | 0.2681 (0.3443, 0.2053) |
| Latvia | 0.0419 (0.0597, 0.0285) | 0.1456 (0.1909, 0.1050) | 0.0034 (0.0045, 0.0025) | 2.2129 (2.9318, 1.5995) | 5.2480 (6.8675, 4.0139) | 0.1426 (0.1885, 0.1097) |
| Lithuania | 0.1062 (0.1458, 0.0752) | 0.5862 (0.7822, 0.4319) | 0.0093 (0.0123, 0.0069) | 4.7820 (6.2906, 3.4901) | 18.1423 (23.4611, 14.1230) | 0.3355 (0.4409, 0.2609) |
| Republic of Moldova | 0.0264 (0.0404, 0.0172) | 0.0919 (0.1160, 0.0738) | 0.0015 (0.0019, 0.0012) | 3.0776 (4.3161, 2.0929) | 8.2945 (11.5155, 6.0237) | 0.1450 (0.2029, 0.1047) |
| Russian Federation | 13.1206 (15.0630, 11.4243) | 99.1982 (108.3223, 89.4310) | 0.0410 (0.0449, 0.0371) | 336.0285 (384.4374, 291.0091) | 2209.8500 (2431.8975, 2007.9376) | 0.9370 (1.0320, 0.8506) |
| Ukraine | 271.8087 (350.0834, 198.3906) | 24.6467 (34.2487, 17.2108) | 0.0303 (0.0421, 0.0209) | 6219.4951 (8033.3356, 4444.3969) | 638.2359 (863.3445, 459.9668) | 0.8201 (1.1111, 0.5902) |
| Brunei Darussalam | 0.0093 (0.0164, 0.0047) | 0.0213 (0.0374, 0.0104) | 0.0082 (0.0145, 0.0042) | 0.5080 (0.7371, 0.3604) | 1.1029 (1.5661, 0.7375) | 0.2849 (0.4253, 0.1891) |
| Japan | 169.7010 (181.5300, 157.4708) | 175.5231 (197.2272, 145.2580) | 0.0347 (0.0386, 0.0295) | 3908.7617 (4181.5634, 3648.6302) | 2743.0831 (3065.0559, 2376.4683) | 0.7123 (0.8055, 0.6215) |
| Republic of Korea | 197.1066 (254.0969, 146.4543) | 347.7714 (453.1629, 266.2407) | 0.3637 (0.4719, 0.2800) | 5223.1491 (6665.9376, 3871.3064) | 6394.8385 (8236.1950, 4946.4788) | 6.6936 (8.5945, 5.1959) |
| Singapore | 0.1744 (0.2025, 0.1460) | 0.2484 (0.3094, 0.2015) | 0.0030 (0.0037, 0.0024) | 7.1978 (9.0070, 5.8197) | 16.6705 (22.5248, 12.0441) | 0.2017 (0.2725, 0.1450) |
| Australia | 21.4039 (24.7168, 18.5242) | 1.8770 (2.3852, 1.4235) | 0.0041 (0.0051, 0.0032) | 438.1810 (506.5497, 377.5416) | 47.4033 (57.2885, 38.2717) | 0.1168 (0.1405, 0.0957) |
| New Zealand | 0.0594 (0.0843, 0.0406) | 0.1249 (0.1847, 0.0780) | 0.0014 (0.0020, 0.0009) | 4.7876 (6.6551, 3.4071) | 5.0833 (6.7987, 3.7846) | 0.0712 (0.0980, 0.0517) |
| Andorra | 0.0218 (0.0459, 0.0072) | 0.0169 (0.0365, 0.0051) | 0.0099 (0.0212, 0.0030) | 0.4416 (0.8307, 0.1817) | 0.2961 (0.5637, 0.1281) | 0.1890 (0.3490, 0.0856) |
| Austria | 4.9848 (5.8777, 4.2373) | 1.4010 (1.6476, 1.1544) | 0.0068 (0.0079, 0.0057) | 129.1454 (152.2018, 109.8181) | 43.4198 (53.1988, 36.0486) | 0.2601 (0.3237, 0.2108) |
| Belgium | 352.8342 (415.8457, 299.3933) | 42.8632 (51.8367, 33.5950) | 0.1446 (0.1741, 0.1148) | 7119.5293 (8308.6746, 6135.5155) | 623.7144 (733.4474, 512.4080) | 2.4079 (2.7934, 2.0105) |
| Cyprus | 1.2662 (2.1528, 0.6579) | 0.8810 (1.3813, 0.5214) | 0.0420 (0.0631, 0.0255) | 24.5661 (39.4879, 13.6559) | 18.7986 (27.0681, 12.4161) | 0.9061 (1.3055, 0.6204) |
| Denmark | 5.0565 (6.2525, 3.9897) | 0.8844 (1.1281, 0.6998) | 0.0068 (0.0085, 0.0055) | 108.5134 (132.3134, 89.3719) | 18.9275 (23.1678, 15.2155) | 0.1757 (0.2134, 0.1430) |
| Finland | 2.1741 (2.7561, 1.6562) | 0.4653 (0.6698, 0.3330) | 0.0032 (0.0045, 0.0023) | 55.2219 (68.3667, 44.4923) | 9.9720 (12.8884, 7.6091) | 0.0973 (0.1300, 0.0740) |
| France | 287.2260 (338.7276, 243.2279) | 24.6991 (29.7853, 19.9196) | 0.0138 (0.0163, 0.0113) | 5737.0173 (6762.6133, 4900.5249) | 405.1546 (472.2812, 334.9701) | 0.2721 (0.3151, 0.2290) |
| Germany | 72.0594 (93.5439, 53.6455) | 14.6806 (18.1903, 11.5413) | 0.0065 (0.0078, 0.0052) | 1461.7349 (1857.0479, 1099.1560) | 263.6596 (314.1380, 218.6286) | 0.1371 (0.1628, 0.1143) |
| Greece | 1.0205 (1.1873, 0.8605) | 0.3574 (0.4374, 0.2892) | 0.0013 (0.0015, 0.0010) | 42.3378 (54.6360, 33.1161) | 27.4505 (37.8391, 18.7152) | 0.1431 (0.1998, 0.0954) |
| Iceland | 0.0684 (0.0813, 0.0577) | 0.0049 (0.0065, 0.0039) | 0.0008 (0.0010, 0.0006) | 1.8912 (2.2510, 1.6114) | 0.6607 (0.9333, 0.4562) | 0.1305 (0.1869, 0.0888) |
| Ireland | 2.3794 (2.8813, 1.9618) | 0.2043 (0.2542, 0.1585) | 0.0024 (0.0030, 0.0019) | 55.1168 (64.5382, 46.8719) | 8.9602 (11.6339, 6.8188) | 0.1277 (0.1706, 0.0946) |
| Israel | 0.7737 (0.9028, 0.6559) | 0.2520 (0.3122, 0.1969) | 0.0019 (0.0023, 0.0015) | 20.5406 (24.0762, 17.0658) | 11.8740 (15.6242, 8.9230) | 0.1051 (0.1413, 0.0771) |
| Italy | 48.9103 (54.2351, 43.9115) | 3.3188 (3.8810, 2.8103) | 0.0020 (0.0023, 0.0017) | 1101.8529 (1218.5396, 991.3799) | 71.7190 (83.5291, 61.3802) | 0.0605 (0.0749, 0.0502) |
| Luxembourg | 0.1925 (0.2391, 0.1551) | 0.0564 (0.0707, 0.0447) | 0.0050 (0.0063, 0.0040) | 4.9913 (6.0275, 4.0917) | 2.0852 (2.6852, 1.6529) | 0.2221 (0.2916, 0.1727) |
| Malta | 0.3050 (0.3630, 0.2522) | 0.0322 (0.0429, 0.0245) | 0.0030 (0.0039, 0.0023) | 8.8567 (10.4923, 7.4286) | 2.4743 (3.4348, 1.7806) | 0.3629 (0.5197, 0.2518) |
| Netherlands | 8.3825 (10.1720, 6.7584) | 1.9833 (2.3545, 1.5889) | 0.0051 (0.0060, 0.0041) | 184.9202 (217.7718, 152.5135) | 44.3942 (53.4536, 36.6585) | 0.1372 (0.1705, 0.1115) |
| Norway | 0.4943 (0.5633, 0.4300) | 0.1198 (0.1704, 0.0807) | 0.0008 (0.0012, 0.0006) | 13.7981 (16.3925, 11.7107) | 3.1943 (4.2347, 2.4100) | 0.0374 (0.0526, 0.0268) |
| Portugal | 3.6492 (4.4251, 3.0038) | 1.1415 (1.3789, 0.9275) | 0.0040 (0.0049, 0.0033) | 78.5066 (93.7933, 65.8213) | 20.7412 (24.9116, 17.2991) | 0.0896 (0.1068, 0.0748) |
| Spain | 8.3135 (9.7825, 7.0237) | 2.5183 (3.1078, 2.0245) | 0.0022 (0.0026, 0.0018) | 189.6610 (219.0874, 164.4338) | 44.5542 (52.4544, 37.7648) | 0.0463 (0.0542, 0.0394) |
| Sweden | 0.8905 (1.0638, 0.7438) | 0.7122 (0.9288, 0.5311) | 0.0030 (0.0039, 0.0023) | 20.8289 (24.5128, 17.4078) | 22.0143 (27.7434, 17.4335) | 0.1237 (0.1603, 0.0959) |
| Switzerland | 0.9566 (1.1730, 0.7549) | 0.2503 (0.3080, 0.2007) | 0.0012 (0.0015, 0.0010) | 24.0631 (29.0133, 19.3650) | 7.5816 (9.4973, 5.9848) | 0.0502 (0.0662, 0.0386) |
| United Kingdom | 269.1216 (280.2897, 253.2381) | 142.9375 (155.8103, 127.2001) | 0.0904 (0.0985, 0.0806) | 4715.4913 (4920.8625, 4472.3292) | 2069.2515 (2248.5940, 1861.0975) | 1.3993 (1.5201, 1.2683) |
| Argentina | 6.3005 (7.8174, 5.0526) | 9.1719 (11.2627, 7.3782) | 0.0158 (0.0194, 0.0128) | 176.7555 (216.8609, 145.8947) | 228.2705 (275.6003, 188.9602) | 0.4098 (0.4947, 0.3391) |
| Chile | 2.3511 (2.7946, 1.9952) | 2.7925 (3.4580, 2.2219) | 0.0106 (0.0132, 0.0085) | 68.6479 (81.0418, 58.5882) | 77.0969 (92.7131, 63.2337) | 0.3069 (0.3708, 0.2507) |
| Uruguay | 0.5432 (0.6483, 0.4433) | 0.3461 (0.4332, 0.2783) | 0.0056 (0.0069, 0.0045) | 16.6461 (19.9742, 13.6076) | 11.2743 (14.1578, 8.8397) | 0.2193 (0.2778, 0.1716) |
| Canada | 7.6169 (9.3240, 6.2177) | 2.2169 (2.9165, 1.6818) | 0.0027 (0.0035, 0.0021) | 149.9357 (177.9338, 125.4268) | 67.4509 (84.6912, 52.3822) | 0.0916 (0.1157, 0.0718) |
| United States of America | 739.2346 (809.1450, 665.3925) | 195.7863 (218.2796, 168.8958) | 0.0309 (0.0343, 0.0267) | 13524.9655 (14696.8341, 12374.8497) | 4349.0413 (4937.2175, 3809.9093) | 0.7291 (0.8344, 0.6376) |
| Antigua and Barbuda | 0.0001 (0.0002, 0.0001) | 0.0000 (0.0000, 0.0000) | 0.0000 (0.0000, 0.0000) | 0.0403 (0.0576, 0.0266) | 0.0405 (0.0592, 0.0256) | 0.0385 (0.0567, 0.0247) |
| Bahamas | 0.0153 (0.0191, 0.0117) | 0.0069 (0.0099, 0.0045) | 0.0019 (0.0027, 0.0013) | 0.5165 (0.6343, 0.4133) | 0.2614 (0.3498, 0.1894) | 0.0642 (0.0853, 0.0469) |
| Barbados | 0.0167 (0.0200, 0.0140) | 0.0109 (0.0145, 0.0082) | 0.0021 (0.0028, 0.0016) | 0.6442 (0.8103, 0.5090) | 0.5473 (0.7088, 0.4071) | 0.1178 (0.1557, 0.0868) |
| Belize | 0.1706 (0.2102, 0.1188) | 0.2280 (0.2792, 0.1872) | 0.0823 (0.1005, 0.0676) | 4.3837 (5.4654, 3.1594) | 6.0793 (7.3657, 4.9853) | 1.9079 (2.3379, 1.5741) |
| Cuba | 0.6904 (0.8351, 0.5583) | 0.0491 (0.0607, 0.0393) | 0.0002 (0.0003, 0.0002) | 25.7469 (32.0777, 20.6486) | 7.1347 (10.2236, 4.6253) | 0.0418 (0.0603, 0.0274) |
| Dominica | 0.0025 (0.0035, 0.0016) | 0.0031 (0.0046, 0.0021) | 0.0039 (0.0056, 0.0026) | 0.1155 (0.1528, 0.0832) | 0.1267 (0.1686, 0.0944) | 0.1542 (0.2036, 0.1162) |
| Dominican Republic | 0.2740 (0.4146, 0.1595) | 0.6869 (1.1210, 0.4123) | 0.0070 (0.0114, 0.0042) | 10.8596 (14.5127, 7.6104) | 23.0119 (34.2942, 15.9334) | 0.2234 (0.3358, 0.1541) |
| Grenada | 0.1486 (0.1759, 0.1155) | 0.0334 (0.0417, 0.0267) | 0.0294 (0.0367, 0.0235) | 3.3893 (3.9475, 2.8502) | 0.9404 (1.1499, 0.7678) | 0.8071 (0.9845, 0.6587) |
| Guyana | 0.0181 (0.0219, 0.0149) | 0.0223 (0.0329, 0.0151) | 0.0037 (0.0054, 0.0026) | 0.9324 (1.1978, 0.7353) | 0.9136 (1.2509, 0.6726) | 0.1351 (0.1834, 0.1014) |
| Haiti | 0.6739 (1.3177, 0.2537) | 0.9042 (1.7769, 0.3394) | 0.0155 (0.0309, 0.0060) | 23.3864 (40.8830, 11.0516) | 31.7500 (57.1538, 14.9264) | 0.4023 (0.7093, 0.1904) |
| Jamaica | 2.4160 (2.9535, 1.9005) | 0.7727 (1.0616, 0.5557) | 0.0247 (0.0342, 0.0179) | 52.6401 (62.7944, 43.0567) | 17.9641 (24.3338, 13.1777) | 0.5839 (0.7919, 0.4274) |
| Saint Lucia | 0.0573 (0.0724, 0.0442) | 0.0124 (0.0158, 0.0095) | 0.0054 (0.0068, 0.0041) | 1.3530 (1.6407, 1.1079) | 0.3965 (0.5010, 0.3193) | 0.1727 (0.2182, 0.1390) |
| Saint Vincent and the Grenadines | 0.0027 (0.0034, 0.0022) | 0.0021 (0.0026, 0.0017) | 0.0016 (0.0020, 0.0013) | 0.1645 (0.2199, 0.1207) | 0.1395 (0.1881, 0.1048) | 0.1024 (0.1377, 0.0769) |
| Suriname | 0.1863 (0.2539, 0.1201) | 0.1185 (0.2128, 0.0690) | 0.0195 (0.0354, 0.0113) | 5.0380 (6.6909, 3.3850) | 3.4249 (5.5807, 2.1823) | 0.5433 (0.9032, 0.3488) |
| Trinidad and Tobago | 0.0876 (0.1042, 0.0732) | 0.0567 (0.0757, 0.0410) | 0.0030 (0.0040, 0.0022) | 2.6646 (3.1595, 2.2297) | 2.0280 (2.6202, 1.5733) | 0.1089 (0.1404, 0.0848) |
| Bolivia (Plurinational State of) | 3.9742 (5.5507, 2.7240) | 8.9207 (13.0030, 5.7069) | 0.1093 (0.1574, 0.0707) | 105.1865 (145.4938, 73.5627) | 211.3781 (303.0630, 139.9151) | 2.3476 (3.3431, 1.5523) |
| Ecuador | 2.7817 (3.1590, 2.4143) | 4.0791 (5.2867, 3.1158) | 0.0262 (0.0338, 0.0201) | 75.8821 (85.8029, 66.3655) | 110.5706 (136.9198, 88.1328) | 0.6751 (0.8355, 0.5383) |
| Peru | 23.1286 (32.3653, 16.5904) | 75.6135 (110.4907, 50.7907) | 0.2295 (0.3353, 0.1537) | 601.6765 (827.5259, 451.2163) | 1625.3465 (2287.3618, 1133.3599) | 4.8400 (6.8241, 3.3595) |
| Colombia | 22.0566 (24.6680, 19.4227) | 47.4146 (58.4867, 38.2500) | 0.0858 (0.1062, 0.0690) | 624.6263 (698.3400, 557.0040) | 1194.5479 (1422.7130, 991.6501) | 2.1713 (2.5873, 1.7996) |
| Costa Rica | 0.2288 (0.2722, 0.1895) | 0.5002 (0.6015, 0.4017) | 0.0090 (0.0108, 0.0072) | 11.2233 (14.3429, 8.7445) | 23.2471 (30.0203, 18.2031) | 0.4221 (0.5460, 0.3302) |
| El Salvador | 0.1105 (0.1602, 0.0703) | 0.1516 (0.2538, 0.0911) | 0.0023 (0.0038, 0.0014) | 15.7564 (22.6530, 10.9336) | 24.7793 (34.9816, 17.1605) | 0.3910 (0.5532, 0.2699) |
| Guatemala | 3.7911 (4.3277, 3.2992) | 5.0368 (6.1168, 4.2042) | 0.0505 (0.0609, 0.0418) | 126.0788 (144.9867, 110.0945) | 187.8536 (228.0599, 155.7594) | 1.6377 (1.9857, 1.3541) |
| Honduras | 1.3452 (2.2049, 0.8116) | 3.9463 (5.7752, 2.5005) | 0.0706 (0.1045, 0.0432) | 45.3395 (69.2667, 30.7571) | 122.4193 (168.6655, 86.7781) | 1.8846 (2.5980, 1.3425) |
| Mexico | 34.5752 (37.0587, 32.1586) | 41.4802 (48.3571, 35.5872) | 0.0351 (0.0409, 0.0302) | 967.2713 (1064.1331, 882.7430) | 1453.0159 (1760.9127, 1228.5462) | 1.1452 (1.3884, 0.9707) |
| Nicaragua | 0.2693 (0.3892, 0.1872) | 0.5577 (0.8385, 0.3498) | 0.0125 (0.0188, 0.0078) | 11.4125 (15.3076, 8.3405) | 24.7286 (33.5110, 18.8673) | 0.4763 (0.6411, 0.3657) |
| Panama | 0.1388 (0.1623, 0.1172) | 0.3013 (0.3840, 0.2314) | 0.0067 (0.0085, 0.0051) | 10.0026 (13.4587, 7.5058) | 23.8269 (32.6823, 17.3708) | 0.5353 (0.7341, 0.3906) |
| Venezuela (Bolivarian Republic of) | 2.5844 (2.9918, 2.1915) | 5.0680 (6.7804, 3.7383) | 0.0181 (0.0242, 0.0133) | 108.7381 (133.8650, 88.8518) | 214.6536 (274.0856, 164.5766) | 0.7276 (0.9247, 0.5607) |
| Brazil | 37.4201 (43.2084, 32.8510) | 120.6107 (134.3944, 107.8763) | 0.0495 (0.0552, 0.0442) | 1310.3951 (1459.9926, 1163.9740) | 3258.8052 (3611.4156, 2961.1797) | 1.3057 (1.4470, 1.1853) |
| Paraguay | 0.5341 (0.7893, 0.3179) | 1.3918 (2.2495, 0.8469) | 0.0249 (0.0400, 0.0151) | 15.1369 (21.2222, 9.3936) | 37.3879 (58.1485, 24.1366) | 0.6164 (0.9639, 0.3993) |
| Algeria | 0.8036 (1.9588, 0.4278) | 6.1762 (10.9727, 3.9885) | 0.0229 (0.0376, 0.0150) | 44.5657 (76.3816, 30.7502) | 204.7180 (335.1107, 144.2354) | 0.5752 (0.9090, 0.4075) |
| Bahrain | 0.0136 (0.0207, 0.0060) | 0.0898 (0.1603, 0.0490) | 0.0155 (0.0279, 0.0082) | 0.5088 (0.7863, 0.3039) | 3.1479 (5.4090, 1.8592) | 0.3174 (0.5581, 0.1849) |
| Egypt | 133.7287 (204.1436, 92.7060) | 165.9094 (238.3968, 115.2173) | 0.3064 (0.4460, 0.2094) | 4096.5746 (6065.8437, 2855.3816) | 4946.6421 (6980.8410, 3452.1909) | 7.1244 (10.1191, 5.0264) |
| Iran (Islamic Republic of) | 3.0918 (4.1947, 2.2311) | 25.5835 (30.5060, 21.4445) | 0.0365 (0.0436, 0.0302) | 159.1791 (203.3004, 121.6714) | 792.0853 (946.1776, 665.6531) | 0.9866 (1.1656, 0.8332) |
| Iraq | 7.0653 (10.2218, 4.8252) | 16.2644 (23.8057, 10.3870) | 0.0748 (0.1123, 0.0479) | 241.7548 (327.6736, 167.9271) | 532.6839 (785.4578, 350.6506) | 1.9076 (2.7333, 1.2891) |
| Jordan | 0.1011 (0.2505, 0.0509) | 0.9940 (1.9394, 0.6118) | 0.0162 (0.0311, 0.0099) | 4.2304 (8.7440, 2.6137) | 34.2724 (62.9501, 22.6266) | 0.4025 (0.7270, 0.2681) |
| Kuwait | 0.0009 (0.0013, 0.0006) | 0.0183 (0.0253, 0.0123) | 0.0007 (0.0010, 0.0004) | 0.0992 (0.1395, 0.0683) | 1.6345 (2.2757, 1.1555) | 0.0357 (0.0481, 0.0261) |
| Lebanon | 3.3227 (4.6375, 2.1889) | 7.8937 (10.5230, 5.7994) | 0.1223 (0.1618, 0.0902) | 100.1050 (139.3214, 67.5171) | 173.7292 (221.1863, 133.1637) | 2.8155 (3.5987, 2.1504) |
| Libya | 0.1129 (0.2722, 0.0528) | 1.1047 (2.4625, 0.5836) | 0.0229 (0.0514, 0.0120) | 6.5471 (10.6979, 4.2928) | 39.8674 (77.9603, 24.1251) | 0.6571 (1.3022, 0.3955) |
| Morocco | 1.0570 (2.8654, 0.4654) | 7.3314 (13.1868, 4.3614) | 0.0244 (0.0434, 0.0150) | 53.9261 (102.3964, 34.2154) | 227.7720 (379.7294, 152.0864) | 0.6601 (1.0971, 0.4414) |
| Palestine | 0.9545 (1.4098, 0.5928) | 2.1507 (3.1894, 1.2685) | 0.1096 (0.1697, 0.0631) | 27.8155 (39.6414, 18.3206) | 64.5176 (90.2582, 42.9191) | 2.3048 (3.3018, 1.4540) |
| Oman | 0.0148 (0.0311, 0.0059) | 0.0407 (0.0878, 0.0161) | 0.0024 (0.0049, 0.0010) | 2.5217 (3.7925, 1.6050) | 7.2800 (11.1782, 4.6508) | 0.1819 (0.2744, 0.1193) |
| Qatar | 0.0124 (0.0272, 0.0058) | 0.2700 (0.4707, 0.1284) | 0.0495 (0.0826, 0.0235) | 0.5744 (1.1033, 0.3561) | 14.9781 (23.3967, 9.4070) | 1.0523 (1.6380, 0.6099) |
| Saudi Arabia | 0.1377 (0.2458, 0.0677) | 1.2147 (1.8185, 0.8119) | 0.0064 (0.0095, 0.0044) | 16.6774 (23.9345, 11.4410) | 105.0245 (142.4419, 74.3144) | 0.3135 (0.4072, 0.2311) |
| Syrian Arab Republic | 0.4629 (1.2826, 0.2341) | 3.1862 (6.1975, 1.7677) | 0.0294 (0.0575, 0.0159) | 23.2912 (44.8878, 15.1549) | 98.9271 (175.2947, 60.1576) | 0.7561 (1.3263, 0.4751) |
| Tunisia | 0.3391 (0.8655, 0.1678) | 2.6778 (4.8721, 1.5146) | 0.0224 (0.0409, 0.0126) | 17.2442 (32.7116, 11.5059) | 76.9269 (126.9911, 53.1204) | 0.5963 (0.9659, 0.4146) |
| Turkey | 16.6737 (27.1318, 9.3889) | 23.7850 (33.9281, 14.3658) | 0.0268 (0.0380, 0.0163) | 572.1410 (952.6863, 371.5089) | 760.7118 (1002.8778, 533.4944) | 0.8160 (1.0723, 0.5734) |
| United Arab Emirates | 0.0456 (0.0949, 0.0149) | 0.6318 (0.9763, 0.3747) | 0.0269 (0.0435, 0.0154) | 3.0204 (4.9298, 1.6959) | 36.9935 (51.4167, 25.1103) | 0.6639 (0.9913, 0.4267) |
| Yemen | 0.5001 (1.0874, 0.2117) | 3.6208 (6.9027, 1.7366) | 0.0289 (0.0565, 0.0139) | 24.5456 (40.2836, 14.8092) | 134.1185 (221.8455, 79.8511) | 0.7786 (1.3628, 0.4458) |
| Afghanistan | 0.9039 (2.4220, 0.3678) | 3.2871 (7.0463, 1.7037) | 0.0371 (0.0837, 0.0182) | 32.8300 (68.9400, 17.9258) | 129.4834 (241.3637, 76.3546) | 0.9822 (1.9586, 0.5648) |
| Bangladesh | 31.6285 (66.5467, 11.6063) | 41.8498 (80.3458, 20.6625) | 0.0340 (0.0660, 0.0176) | 879.4821 (1634.8960, 384.3750) | 1063.2727 (1781.7766, 630.1669) | 0.7677 (1.3123, 0.4451) |
| Bhutan | 0.1077 (0.2225, 0.0448) | 0.2286 (0.4413, 0.1190) | 0.0422 (0.0815, 0.0221) | 3.3600 (6.2339, 1.7831) | 5.2042 (9.0660, 3.1492) | 0.8610 (1.5266, 0.5170) |
| India | 301.7225 (557.0883, 143.3142) | 604.2741 (896.0656, 383.4427) | 0.0579 (0.0852, 0.0370) | 8294.3421 (14317.0583, 4407.5499) | 14559.2552 (20984.6505, 9715.2391) | 1.2417 (1.7911, 0.8203) |
| Nepal | 8.0161 (15.9351, 2.9223) | 14.1912 (26.6073, 7.1693) | 0.0716 (0.1309, 0.0363) | 229.7714 (409.7448, 99.7450) | 392.4573 (648.9321, 236.3293) | 1.6690 (2.7929, 0.9824) |
| Pakistan | 29.8984 (55.8885, 14.4252) | 38.7223 (61.0613, 22.3830) | 0.0411 (0.0644, 0.0246) | 778.3987 (1300.9317, 424.4947) | 1094.1485 (1613.3156, 713.0382) | 0.8947 (1.3347, 0.5612) |
| Angola | 5.2379 (8.4402, 3.1919) | 6.9194 (11.6053, 3.9024) | 0.0762 (0.1269, 0.0410) | 148.9996 (243.9262, 89.3643) | 190.2410 (314.1558, 111.2198) | 1.6107 (2.6644, 0.9274) |
| Central African Republic | 2.4342 (4.3129, 1.0959) | 2.8597 (5.8738, 0.9931) | 0.1607 (0.3392, 0.0580) | 70.1119 (125.4187, 33.3787) | 85.0793 (175.9838, 29.3922) | 3.6352 (7.5164, 1.3199) |
| Congo | 1.7722 (3.1277, 0.8480) | 2.0921 (3.5775, 1.1336) | 0.0997 (0.1735, 0.0532) | 47.9438 (82.2913, 23.3937) | 56.4092 (97.9428, 31.5713) | 2.0864 (3.5623, 1.1596) |
| Democratic Republic of the Congo | 23.1077 (41.7677, 10.6966) | 41.0404 (86.6336, 12.8749) | 0.1422 (0.3262, 0.0423) | 622.1832 (1110.6961, 292.4604) | 1093.2390 (2235.5147, 350.6260) | 2.9974 (6.3196, 0.9701) |
| Equatorial Guinea | 0.3786 (0.7028, 0.1801) | 0.2457 (0.4603, 0.1267) | 0.0607 (0.1118, 0.0317) | 10.4814 (19.4282, 4.9997) | 6.6799 (11.8422, 3.4711) | 1.2680 (2.3461, 0.6799) |
| Gabon | 0.6936 (1.1512, 0.3471) | 0.5804 (1.0893, 0.2934) | 0.0680 (0.1262, 0.0344) | 16.8467 (28.0162, 9.1190) | 14.7597 (28.1897, 7.8822) | 1.4254 (2.6585, 0.7564) |
| Burundi | 4.1318 (7.0045, 1.9490) | 3.4910 (7.1636, 1.3989) | 0.0897 (0.1817, 0.0363) | 105.6859 (180.1302, 52.9185) | 92.9705 (187.4390, 39.7233) | 1.8922 (3.8570, 0.8056) |
| Comoros | 0.2810 (0.4889, 0.1336) | 0.3265 (0.5790, 0.1534) | 0.0793 (0.1430, 0.0378) | 7.1453 (12.0903, 3.4857) | 7.7724 (12.9589, 3.7297) | 1.6193 (2.7554, 0.7939) |
| Djibouti | 0.1363 (0.2406, 0.0725) | 0.3293 (0.7332, 0.1296) | 0.0679 (0.1491, 0.0282) | 3.8765 (6.7497, 2.0377) | 9.1178 (20.2322, 3.7996) | 1.4381 (3.0891, 0.6162) |
| Eritrea | 1.5066 (2.7021, 0.7542) | 1.7126 (2.9591, 1.0357) | 0.0763 (0.1287, 0.0441) | 48.8584 (88.8294, 25.3939) | 50.3374 (88.7894, 29.7628) | 1.6988 (2.8745, 1.0572) |
| Ethiopia | 24.2321 (44.6948, 10.6374) | 18.1009 (30.0506, 10.5490) | 0.0494 (0.0814, 0.0287) | 680.0721 (1259.6942, 314.9874) | 446.7116 (753.1773, 262.9710) | 1.0217 (1.7098, 0.6095) |
| Kenya | 8.1596 (14.9974, 3.7427) | 17.0886 (37.7243, 7.7805) | 0.0937 (0.2311, 0.0413) | 200.0293 (340.7048, 94.2128) | 431.4042 (849.1234, 206.6890) | 1.9122 (4.1229, 0.8896) |
| Madagascar | 9.4681 (15.3900, 5.5489) | 11.8339 (21.5615, 5.8737) | 0.1461 (0.2691, 0.0719) | 240.8777 (386.0591, 143.9171) | 331.1007 (587.5320, 176.2844) | 2.9349 (5.2655, 1.4894) |
| Malawi | 4.3879 (8.0411, 2.1218) | 5.8098 (11.4625, 2.6868) | 0.0938 (0.1781, 0.0426) | 112.2659 (199.4502, 55.4658) | 152.9626 (304.2148, 73.1878) | 2.0219 (3.9660, 0.9606) |
| Mauritius | 0.0214 (0.0243, 0.0187) | 0.0540 (0.0632, 0.0449) | 0.0032 (0.0038, 0.0027) | 1.7004 (2.3459, 1.2296) | 2.4915 (3.2156, 1.9321) | 0.1436 (0.1857, 0.1120) |
| Mozambique | 5.7843 (11.3221, 2.6578) | 7.7931 (14.3722, 3.7652) | 0.0828 (0.1475, 0.0408) | 150.7230 (287.3771, 69.6734) | 218.2545 (406.6802, 108.0215) | 1.8573 (3.3875, 0.9238) |
| Rwanda | 4.8032 (8.2332, 2.5770) | 3.9062 (6.9841, 1.9685) | 0.0772 (0.1410, 0.0387) | 129.2920 (221.8812, 71.3196) | 99.2482 (170.2664, 52.8586) | 1.5901 (2.7742, 0.8382) |
| Seychelles | 0.0022 (0.0036, 0.0013) | 0.0020 (0.0041, 0.0011) | 0.0021 (0.0043, 0.0011) | 0.0986 (0.1323, 0.0717) | 0.1199 (0.1710, 0.0827) | 0.1063 (0.1513, 0.0743) |
| Somalia | 4.7498 (11.5541, 1.3017) | 6.5080 (19.5218, 1.2739) | 0.1260 (0.3742, 0.0244) | 139.6555 (346.4690, 39.7363) | 196.4152 (577.5738, 41.9582) | 2.9435 (8.4752, 0.6441) |
| United Republic of Tanzania | 10.6273 (19.1414, 5.7308) | 12.1451 (20.8238, 6.8952) | 0.0559 (0.0952, 0.0314) | 273.8607 (486.1842, 149.5995) | 307.0935 (520.2617, 179.3971) | 1.1848 (1.9593, 0.6996) |
| Uganda | 9.8170 (21.2640, 3.8190) | 10.5637 (20.2719, 4.6525) | 0.0863 (0.1665, 0.0380) | 245.2416 (529.8073, 98.9844) | 273.3742 (514.3585, 125.8107) | 1.8165 (3.4069, 0.8273) |
| Zambia | 3.9380 (7.1572, 2.0373) | 5.4267 (9.6053, 3.0124) | 0.0930 (0.1652, 0.0523) | 102.9554 (182.2319, 53.7717) | 149.1502 (270.7812, 86.4555) | 2.0450 (3.5865, 1.1575) |
| Botswana | 1.4283 (2.6608, 0.7790) | 1.8080 (3.7331, 0.8602) | 0.1517 (0.3079, 0.0711) | 36.4364 (67.2581, 20.2156) | 45.4277 (92.1249, 22.1179) | 3.1222 (6.2761, 1.5275) |
| Lesotho | 2.2944 (4.5008, 1.0034) | 2.2248 (4.3974, 1.0346) | 0.2375 (0.4770, 0.1134) | 51.8167 (97.2033, 23.4419) | 58.7871 (112.9183, 28.3415) | 5.3264 (10.3093, 2.5755) |
| Namibia | 1.4507 (2.6032, 0.8196) | 1.5865 (2.7243, 0.9476) | 0.1444 (0.2440, 0.0853) | 35.8873 (62.4662, 20.9782) | 38.3016 (65.8944, 23.3072) | 2.8887 (4.8690, 1.7718) |
| South Africa | 25.5581 (38.2804, 18.4345) | 24.2837 (42.3679, 17.0346) | 0.0602 (0.1067, 0.0419) | 688.4427 (980.3674, 512.0276) | 603.0355 (1034.2188, 432.9072) | 1.2845 (2.2240, 0.9154) |
| Eswatini | 1.9049 (4.2203, 0.8501) | 1.5633 (3.2669, 0.7725) | 0.3270 (0.7434, 0.1622) | 49.1353 (107.2450, 22.5396) | 43.4000 (90.8500, 21.7515) | 7.3512 (15.3147, 3.6999) |
| Zimbabwe | 3.4391 (5.5375, 2.0059) | 6.4728 (11.2490, 3.6635) | 0.1156 (0.1949, 0.0666) | 86.3014 (138.6651, 50.6171) | 178.5076 (301.3223, 104.4459) | 2.5562 (4.2933, 1.4846) |
| Benin | 4.9327 (8.9028, 2.2070) | 4.7042 (8.1440, 2.3308) | 0.1107 (0.1921, 0.0548) | 111.0827 (204.9771, 51.4909) | 112.7004 (192.6920, 56.0064) | 2.1968 (3.7560, 1.0883) |
| Burkina Faso | 5.9370 (10.9782, 2.4805) | 6.3693 (12.6924, 3.4069) | 0.0843 (0.1677, 0.0452) | 142.8160 (261.1015, 61.4938) | 152.5115 (306.0659, 81.2859) | 1.6724 (3.3411, 0.9101) |
| Cameroon | 10.2301 (17.0552, 5.5894) | 11.8399 (21.8358, 6.1596) | 0.1140 (0.2024, 0.0610) | 253.6425 (423.3286, 137.1331) | 310.5341 (584.9910, 164.8798) | 2.3932 (4.4263, 1.2432) |
| Cabo Verde | 0.5995 (1.0676, 0.2795) | 0.3137 (0.6050, 0.1567) | 0.0756 (0.1480, 0.0374) | 12.1432 (21.3743, 5.7373) | 6.6661 (13.4316, 3.4913) | 1.5055 (3.0350, 0.7744) |
| Chad | 6.9268 (12.9798, 2.6260) | 7.0956 (15.1709, 2.6071) | 0.1509 (0.3084, 0.0553) | 155.6306 (295.4718, 57.4129) | 176.7549 (383.6906, 66.7805) | 3.0734 (6.6611, 1.1431) |
| C么te d'Ivoire | 8.0505 (14.8137, 3.9755) | 9.8234 (17.3605, 5.2604) | 0.1101 (0.1911, 0.0592) | 217.6124 (397.7198, 109.4575) | 260.9882 (466.6903, 139.2443) | 2.2792 (3.9906, 1.2282) |
| Gambia | 0.7624 (1.5318, 0.3156) | 1.2530 (2.5761, 0.5145) | 0.1495 (0.2986, 0.0609) | 19.3469 (39.7540, 7.8994) | 30.7441 (64.2314, 13.0341) | 3.0913 (6.4152, 1.2993) |
| Ghana | 8.2387 (13.0737, 4.7069) | 14.2783 (23.8964, 8.1622) | 0.1078 (0.1812, 0.0625) | 209.5305 (332.1264, 116.8352) | 351.9282 (577.1298, 200.5217) | 2.1366 (3.5062, 1.2249) |
| Guinea | 8.2084 (14.0875, 3.6331) | 7.3488 (13.5819, 3.3775) | 0.1510 (0.2698, 0.0701) | 185.9414 (319.2612, 79.9655) | 176.4015 (331.3437, 81.5480) | 3.1127 (5.8232, 1.4314) |
| Guinea-Bissau | 1.2732 (2.4616, 0.5077) | 0.9740 (1.9302, 0.4106) | 0.1666 (0.3318, 0.0710) | 33.1505 (64.2073, 13.6305) | 27.5421 (54.4510, 11.4996) | 3.6088 (7.0821, 1.5556) |
| Liberia | 2.5038 (4.3235, 1.1069) | 2.2126 (4.4116, 0.8045) | 0.1309 (0.2551, 0.0487) | 57.6187 (97.1271, 25.5889) | 57.1041 (118.3640, 20.8678) | 2.6387 (5.1650, 0.9703) |
| Mali | 12.2148 (21.8257, 5.5478) | 16.0618 (30.3653, 8.6525) | 0.2222 (0.4267, 0.1218) | 309.4358 (547.5573, 141.6866) | 410.4411 (768.6910, 225.3234) | 4.6022 (8.6604, 2.5197) |
| Mauritania | 2.0776 (3.6059, 1.0502) | 1.6463 (3.1531, 0.8648) | 0.0906 (0.1708, 0.0468) | 48.8001 (86.0138, 24.2844) | 37.1246 (70.0408, 20.3461) | 1.7545 (3.3680, 0.9613) |
| Niger | 5.8943 (11.5551, 1.8621) | 8.3419 (17.8759, 2.9392) | 0.1365 (0.2850, 0.0480) | 149.3864 (292.7053, 51.1612) | 200.6520 (420.5832, 70.5022) | 2.5767 (5.3825, 0.9116) |
| Nigeria | 65.7560 (111.5252, 35.0648) | 55.6565 (92.3074, 35.7746) | 0.0789 (0.1323, 0.0507) | 1508.9823 (2634.5283, 768.9535) | 1290.7804 (2113.4852, 839.0129) | 1.4900 (2.4595, 0.9673) |
| Sao Tome and Principe | 0.2480 (0.4446, 0.1271) | 0.2414 (0.4735, 0.1054) | 0.2646 (0.5133, 0.1168) | 5.3591 (9.7934, 2.6786) | 5.6654 (11.1524, 2.4278) | 5.2165 (10.3210, 2.2920) |
| Senegal | 7.2931 (12.9176, 3.5257) | 7.5588 (13.6166, 4.1336) | 0.1162 (0.2081, 0.0633) | 175.0684 (312.4388, 85.8509) | 177.0590 (319.1531, 95.3151) | 2.3098 (4.1387, 1.2800) |
| Sierra Leone | 5.2518 (9.5704, 2.3435) | 4.1276 (8.0352, 1.6453) | 0.1298 (0.2438, 0.0536) | 119.3450 (220.3883, 55.1446) | 101.7729 (201.3700, 42.0402) | 2.6539 (5.2148, 1.0923) |
| Togo | 2.9470 (5.1275, 1.3612) | 4.4373 (8.4531, 1.9930) | 0.1435 (0.2661, 0.0668) | 74.5839 (127.7910, 35.2536) | 118.9084 (224.7025, 54.0559) | 3.0389 (5.7036, 1.3898) |
| American Samoa | 0.0005 (0.0012, 0.0003) | 0.0013 (0.0029, 0.0006) | 0.0044 (0.0091, 0.0019) | 0.0436 (0.0645, 0.0292) | 0.0789 (0.1174, 0.0528) | 0.1934 (0.2943, 0.1297) |
| Bermuda | 0.0066 (0.0088, 0.0049) | 0.0001 (0.0001, 0.0001) | 0.0001 (0.0001, 0.0001) | 0.1604 (0.2006, 0.1275) | 0.0321 (0.0468, 0.0203) | 0.0295 (0.0427, 0.0189) |
| Cook Islands | 0.0003 (0.0006, 0.0001) | 0.0006 (0.0016, 0.0002) | 0.0027 (0.0075, 0.0009) | 0.0218 (0.0305, 0.0146) | 0.0382 (0.0572, 0.0257) | 0.1581 (0.2377, 0.1056) |
| Greenland | 0.0021 (0.0045, 0.0008) | 0.0025 (0.0063, 0.0009) | 0.0055 (0.0146, 0.0020) | 0.1067 (0.1583, 0.0689) | 0.1399 (0.2142, 0.0879) | 0.2420 (0.3868, 0.1515) |
| Guam | 0.0007 (0.0014, 0.0004) | 0.0018 (0.0039, 0.0008) | 0.0006 (0.0013, 0.0003) | 0.1278 (0.1870, 0.0859) | 0.2635 (0.3823, 0.1733) | 0.1313 (0.1896, 0.0854) |
| Monaco | 0.0027 (0.0048, 0.0012) | 0.0018 (0.0030, 0.0009) | 0.0016 (0.0027, 0.0008) | 0.0706 (0.1058, 0.0445) | 0.0442 (0.0659, 0.0293) | 0.0511 (0.0746, 0.0343) |
| Nauru | 0.0001 (0.0002, 0.0001) | 0.0002 (0.0006, 0.0001) | 0.0074 (0.0204, 0.0024) | 0.0105 (0.0146, 0.0074) | 0.0140 (0.0217, 0.0095) | 0.2989 (0.4865, 0.1978) |
| Niue | 0.0001 (0.0003, 0.0001) | 0.0001 (0.0002, 0.0000) | 0.0046 (0.0113, 0.0020) | 0.0043 (0.0063, 0.0028) | 0.0041 (0.0061, 0.0028) | 0.2087 (0.3090, 0.1403) |
| Northern Mariana Islands | 0.0018 (0.0032, 0.0010) | 0.0041 (0.0093, 0.0011) | 0.0171 (0.0385, 0.0039) | 0.0815 (0.1224, 0.0530) | 0.1371 (0.2379, 0.0785) | 0.3950 (0.6976, 0.2111) |
| Palau | 0.0003 (0.0005, 0.0001) | 0.0005 (0.0010, 0.0002) | 0.0041 (0.0085, 0.0020) | 0.0190 (0.0269, 0.0134) | 0.0393 (0.0561, 0.0267) | 0.2447 (0.3419, 0.1680) |
| Puerto Rico | 0.0861 (0.1098, 0.0697) | 0.0084 (0.0123, 0.0054) | 0.0001 (0.0002, 0.0001) | 3.7921 (4.8385, 2.9479) | 0.8939 (1.2622, 0.6045) | 0.0160 (0.0228, 0.0108) |
| Saint Kitts and Nevis | 0.0125 (0.0148, 0.0106) | 0.0046 (0.0076, 0.0032) | 0.0072 (0.0111, 0.0053) | 0.2866 (0.3348, 0.2451) | 0.1364 (0.2086, 0.0972) | 0.1899 (0.2819, 0.1398) |
| San Marino | 0.0403 (0.0654, 0.0244) | 0.0326 (0.0623, 0.0160) | 0.0348 (0.0675, 0.0174) | 0.7171 (1.1418, 0.4659) | 0.5612 (0.9854, 0.3203) | 0.7415 (1.2741, 0.4347) |
| Tokelau | 0.0000 (0.0001, 0.0000) | 0.0001 (0.0001, 0.0000) | 0.0043 (0.0107, 0.0018) | 0.0024 (0.0036, 0.0017) | 0.0032 (0.0047, 0.0021) | 0.2158 (0.3217, 0.1438) |
| Tuvalu | 0.0002 (0.0005, 0.0001) | 0.0004 (0.0008, 0.0001) | 0.0054 (0.0130, 0.0021) | 0.0135 (0.0200, 0.0088) | 0.0228 (0.0349, 0.0152) | 0.2562 (0.3856, 0.1713) |
| United States Virgin Islands | 0.0004 (0.0007, 0.0002) | 0.0003 (0.0005, 0.0001) | 0.0002 (0.0003, 0.0001) | 0.0560 (0.0798, 0.0381) | 0.0666 (0.0952, 0.0445) | 0.0439 (0.0623, 0.0296) |
| South Sudan | 4.2309 (9.0457, 1.9613) | 3.4244 (7.3187, 1.4303) | 0.1109 (0.2375, 0.0458) | 103.8689 (218.4534, 50.2270) | 90.9466 (186.2257, 40.2901) | 2.3504 (4.9525, 1.0221) |
| Sudan | 1.0018 (2.0472, 0.3133) | 4.7497 (8.6394, 2.2836) | 0.0262 (0.0471, 0.0129) | 46.6179 (79.8958, 25.1443) | 182.1995 (294.1789, 103.2316) | 0.7325 (1.1962, 0.4151) |
